# Platinum-based phosphorescent lifetime probes for the visualisation of G-quadruplex DNA in cells

**DOI:** 10.1039/d5sc08064a

**Published:** 2026-02-04

**Authors:** Adinarayana Bellamkonda, Petr S. Sherin, Timothy Kench, Marina K. Kuimova, Ramon Vilar

**Affiliations:** a Department of Chemistry, Imperial College London White City Campus London W12 0BZ UK m.kuimova@imperial.ac.uk r.vilar@imperial.ac.uk; b Institute of Chemical Biology, Imperial College London, White City Campus London W12 0BZ UK

## Abstract

DNA can fold into a range of different structures besides the canonical double helix. These structures have been shown to play important biological regulatory roles, highlighting that is not only DNA's sequence but also its structure that dictates its functions. However, detecting and visualising these structures in cells is challenging, due to their dynamic nature and low abundance at any one time, as compared to duplex DNA. In this paper we report the syntheses of three new platinum(ii) complexes, coordinated to C^N^N^C and N^C^C^N ligands, and study their photophysical properties in the absence and presence of duplex and quadruplex DNA structures. We find that two of the probes switch on their phosphorescence intensity upon interaction with DNA. Moreover, we demonstrate that the phosphorescence lifetime of one of the probes shows distinct changes upon interaction with quadruplex DNA, as compared to duplex DNA or free in solution. Reassuringly, this probe shows no self-aggregation in the nuclei and nucleoli of live and fixed cells, allowing artefact-free imaging. Thus, we utilise Phosphorescence Lifetime Imaging Microscopy (PLIM) to visualise G-quadruplexes in live and fixed cells using this novel PLIM probe.

## Abstract 2

We report a Pt(ii)-based optical probe with distinct phosphorescence lifetime to visualise G-quadruplex DNA in cells using phosphorescence lifetime imaging microscopy (PLIM).

## Graphical abstract

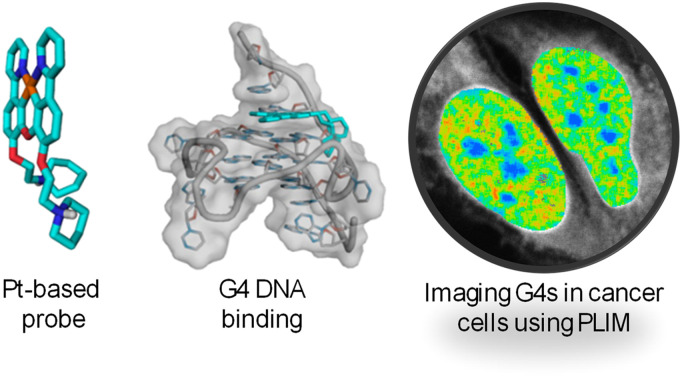

## Introduction

While duplex is the predominant structure of DNA *in vivo*, during cellular processes that require unfolding of the double helix – *e.g.* replication and transcription – DNA can form alternative structures such as 3- and 4-way junctions, *i*-motifs, triplexes and quadruplexes.^1^ The regulatory functions of these non-canonical structures in biological processes continue to emerge, highlighting that not only the sequence of DNA is important, but also its topology.^2,3^ Amongst these structures, guanine-quadruplex (G4) DNA has attracted significant attention since it has been implicated in various cellular functions across species, including regulation of gene expression, telomere maintenance and control of replication.^4–7^ G4s form in guanine-rich sequences thanks to the ability of guanines to self-assemble into tetrads *via* Hoogsteen hydrogen bonding; when two or more of these tetrads assemble in the same run of DNA, they form the G-quadruplex helical arrangement.^8^

Due to G4s′ important biological functions, there has been a continued interest in detecting and visualising them in cells *via* microscopy. The two main approaches to do this are (i) immunostaining with G4-selective antibodies,^9,10^ and (ii) the use of luminescent probes based on small molecules that bind G4s.^11,12^ The former has allowed to unambiguously detect G4 structures in cells as well as enabling important sequencing studies to establish their genomic distribution.^13^ However, one limitation of antibody-based imaging strategies is their reliance on cell fixation and membrane permeabilization, which precludes the studies of G4 dynamics in live cells. To address this, a recent report has shown that a G4-selective nanobody can be expressed intracellularly and used to image endogenous G-quadruplexes in live cells.^14^

In contrast to antibodies, small-molecule fluorescent or phosphorescent probes are generally easier to produce synthetically and can be designed to be cell permeable, allowing for G4 imaging in their native cellular environment providing some insights into their dynamics.^15–17^ Most small-molecule G4 probes rely on emission intensity ‘switch-on’ upon DNA binding. While this has been extensively used for *in vitro* studies, reliable cellular visualisation and imaging of G4s with this type of emitting compounds is challenging. This is because emission intensity is dependent on the probe's concentration and therefore differences in cellular localisation/accumulation can lead to unreliable detection of G4 levels. In addition, unless the probe is highly selective for G4s, it is difficult to differentiate when it is bound to G4 over others (mainly the predominant duplex DNA). To overcome these limitations, alternative strategies have been explored including probes that rely on emission lifetime, which is concentration independent;^18,19^ spectral ratiometric probes, which display significant shifts in their emission wavelengths upon G4 binding;^20–22^ and probes suitable for single-molecule microscopy.^23^

We have focused on the design and development of emission lifetime probes to image G4s in cells. We first reported the use of trianguleniums as efficient fluorescence lifetime-based probes and were able to visualise G4s in live U2OS cells using fluorescence lifetime imaging microscopy (FLIM).^19^ This also allowed us to monitor G4 dynamics by downregulating G4-specifc helicases.^24^ Subsequently we used commercially available probes – thioflavin-T (ThT) and thiazole orange (TO) – as well as derivatives of TO, to visualize both G4 DNA and RNA.^25,26^ Others have developed fluorescence lifetime probes to image G4s in cells, such as the bis-vinylpyridinium carbazole derivative *o*-BMVC,^18,27,28^ and the cationic tripodal dye NBTE,^29^ based on a functionalised tris(4-(pyridin-4-yl) phenyl) amine. While all these fluorescence lifetime probes have provided new insights into G4s, there is interest in improving their dynamic range, *i.e.* the range of emission lifetimes that can be accurately detected and resolved, affinity/selectivity for G4s and brightness.

**Fig. 1:**
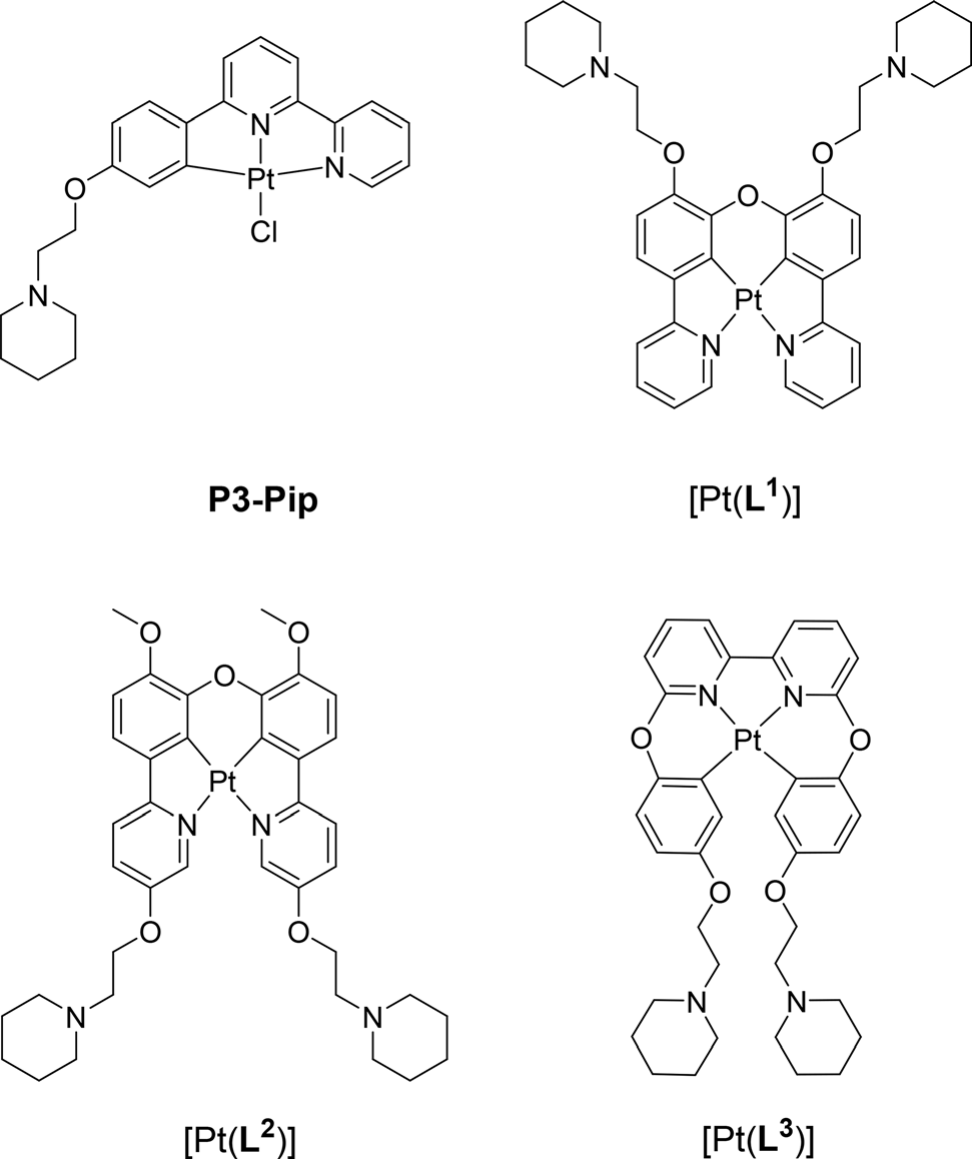
Chemical structure of G4 DNA optical probes based on cyclometallated platinum(ii) complexes previously reported (P3-Pip)^34^ and investigated in this study [Pt(L*^n^*)] (*n* = 1–3).

Cyclometallated platinum(ii) complexes coordinated to polyaromatic ligands have been studied as G4 DNA binders since their square planar geometry and extended aromatic systems are optimal for end-stacking interactions with the G-tetrads.^30,31^ Furthermore, upon DNA binding, this type of complexes tends to switch-on their phosphorescence intensity, making them suitable as DNA optical probes.^32,33^ However, as discussed above, while switch-on probes show excellent performance in *in vitro* studies, their use for cellular imaging is more challenging. To address this issue, recently, we reported the first example of platinum(ii) phosphorescence lifetime probe for G4s (see P3-Pip complex in Fig. 1).^34^ We showed that upon binding to G4 DNA this probe has significantly longer phosphorescence lifetime, allowing to detect the levels of G4s *via* phosphorescence lifetime imaging microscopy (PLIM), in fixed cells. However, the chloride in the 4th coordinating position of the complex, could potentially be exchanged for other ligands in the cellular environment; this in turn could lead to off-target interactions and difficulties in defining the emissive species in cells.^35^ Additionally, we detected a significant prevalence of aggregation of this complex at higher concentration, clearly visible in cellular imaging, presumably due to the dye accumulation. The aggregation manifesting itself in a red shifted Pt⋯Pt MMLCT emission band and a shortening of phosphorescence lifetimes, complicating the PLIM data analysis.

With the aim of developing better optical probes for cellular imaging of G4s *via* PLIM, in this work we report three new platinum(ii) complexes, with tetradentate N^C^C^N and C^N^N^C ligands (Fig. 1). We show that two of these complexes, [Pt(L^1^)] and [Pt(L^2^)], exhibit a switch-on phosphorescence response upon binding to DNA, and longer phosphorescence lifetimes when bound to DNA *vs.* free in solution. More importantly, we have found that [Pt(L^1^)] exhibits significantly longer phosphorescence lifetime when bound to G4 DNA structures as compared to duplex DNA. This distinctive behaviour enabled us to perform PLIM studies with [Pt(L^1^)] in cells to visualize G4 structures, unobstructed by the dye aggregation.

## Result and discussion

### Synthesis and characterisation of [Pt(L*^n^*)]

The three platinum(ii) complexes with cyclometallating ligands were designed as potential G4 DNA binders and optical probes, based on the following criteria: (i) tetradentate ligands to prevent the resulting platinum(ii) complexes from undergoing unwanted substitution reactions; (ii) polyaromatic ligands with the right dimensions for end-stacking with the G-tetrads *via* π–π interactions; in the case of [Pt(L^1^)] and [Pt(L^2^)] the core of the complexes is planar, as has been shown in previous platinum(ii) complexes with analogous N^C^C^N ligands.^36,37^ On the other hand, for [Pt(L^3^)], due to steric hindrance between two of the rings, the complex is not completely planar as previously shown for platinum(ii) complexes with similar C^N^N^C coordination motifs;^38,39^ this could provide higher selectivity for G4 over duplex DNA (an analogous idea using organic helicenes has been previously reported^40^); (iii) introduction of protonatable amines as substituents to increase water solubility of the planar systems and enhance interactions with the negatively charged phosphate backbone of DNA; in addition, these substituents could provide higher selectivity for G4 *vs.* duplex DNA by preventing the complexes from intercalating in between bases of duplex DNA.

**Scheme 1:**
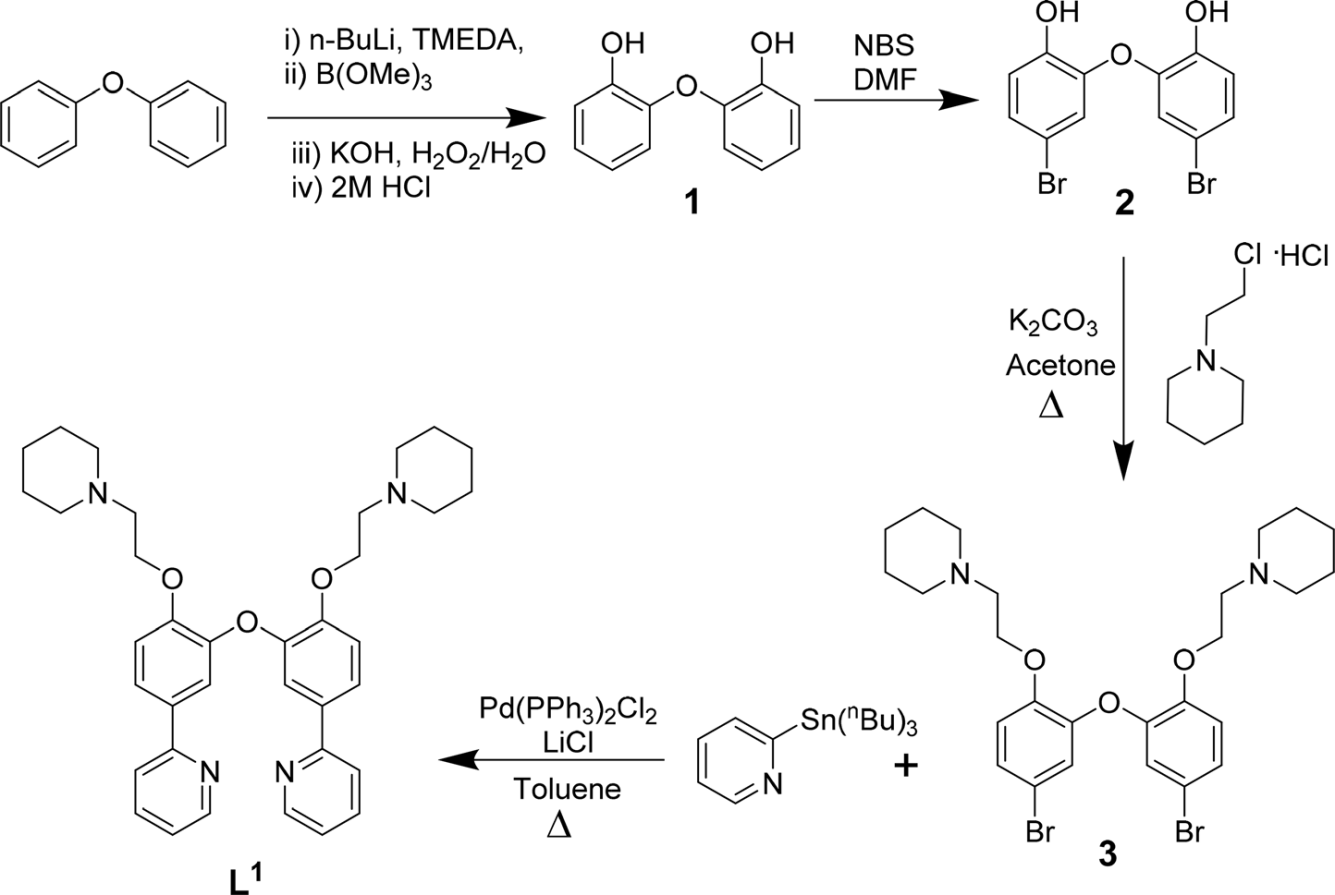
Reaction scheme for the synthesis of L^1^ based on previously reported procedures for other N^C^C^N ligands.^37^

**Scheme 2:**
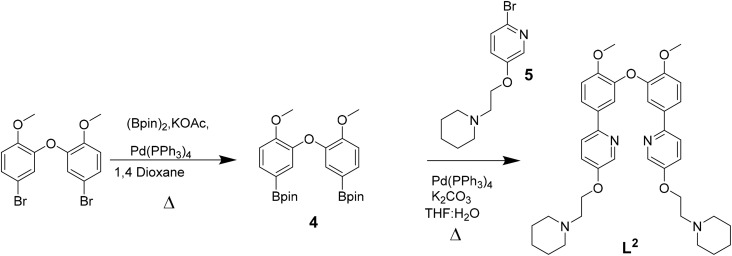
Reaction scheme for the synthesis of L^2^.

**Scheme 3:**
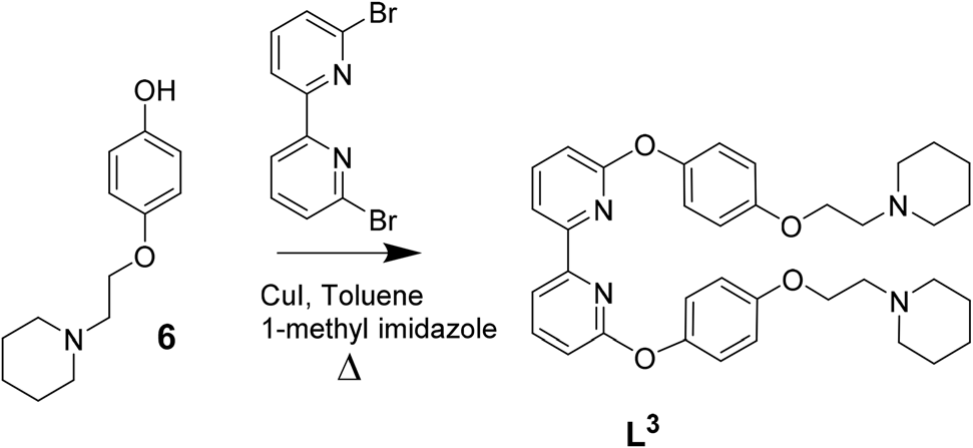
Reaction scheme for the synthesis of L^3^ based on previously reported procedures for other C^N^N^C ligands.^39^

Ligands L^1^–L^3^ were synthesised as shown in Schemes 1–3. The final ligands and all the intermediates were characterised by ^1^H and ^13^C NMR spectroscopy, and mass spectrometry (see SI for details). The purity of the final compounds, was confirmed by LC-MS.

**Scheme 4:**
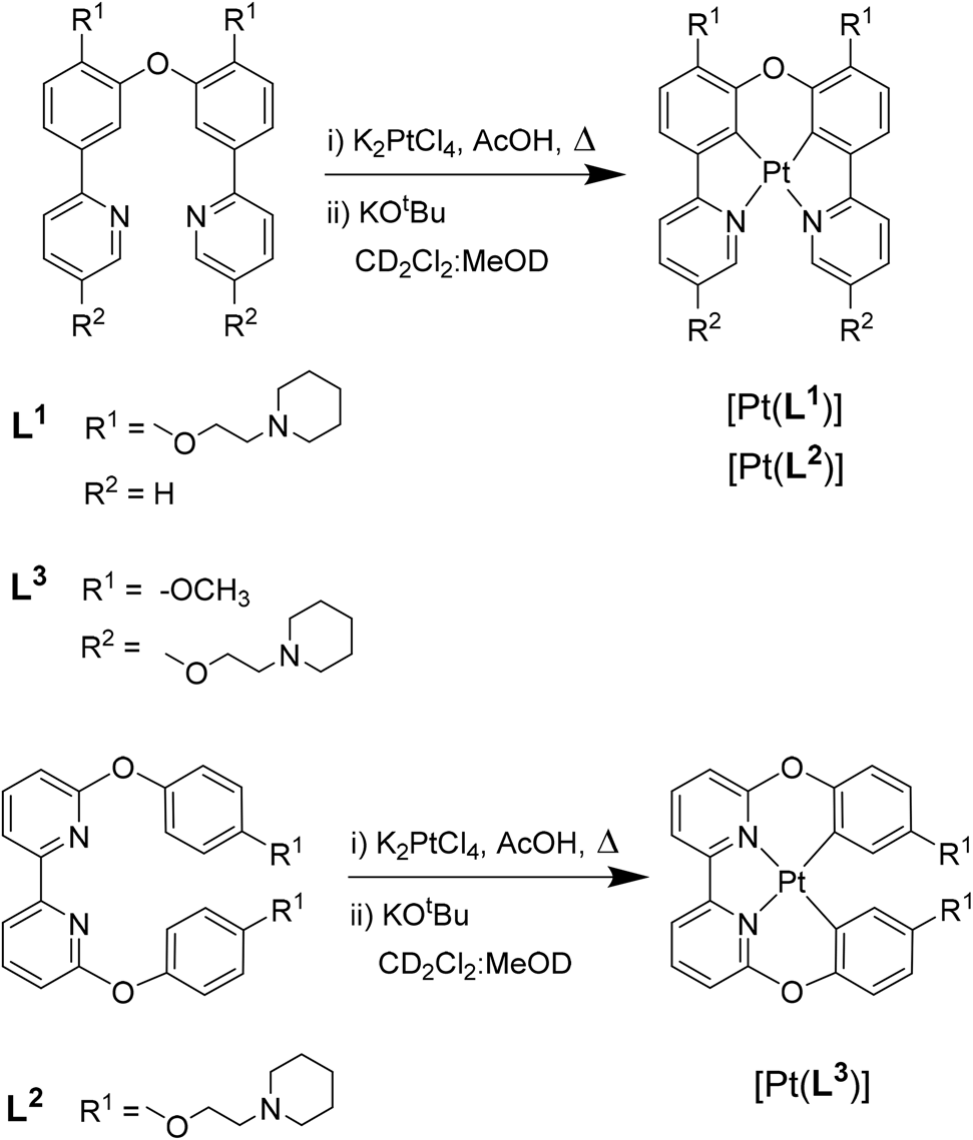
Reaction scheme for the synthesis of platinum(ii) complexes [Pt(L*^n^*)] (*n* = 1, 2, 3).

We then proceeded to the coordination of platinum(ii) to the three new tetradentate ligands, which involved cyclometallating two of the aromatic rings (Scheme 4). After purifying and isolating [Pt(L*^n^*)] (*n* = 1, 2, 3), these three new complexes were characterised by ^1^H, ^13^C and ^195^Pt NMR spectroscopies. In particular, the disappearance of the two aromatic protons in the ^1^H NMR spectra of the complexes, was diagnostic that the reaction had occurred; in addition, we observed the expected shifts of other signals – particularly those closer to the coordinating groups – and the correct integration (see SI for spectroscopic data). The ^195^Pt NMR spectra of the three purified complexes were also consistent with the formation of cyclometallated platinum(ii) complexes, as compared to data previously reported for other tetradentate C^N^N^C and N^C^C^N ligands. The formulation of complexes was further confirmed by mass spectrometry which showed peaks at 772, 832 and 788 a.m.u. for [Pt(L^1^)], [Pt(L^2^)] and [Pt(L^3^)] respectively.

### Photophysical characterisation and DNA binding studies

With the three new platinum(ii) complexes in hand, we proceeded to study their photophysical properties in the absence and presence of DNA. The UV-vis spectra of the three complexes were recorded in CH_2_Cl_2_, CH_3_OH, CH_3_CN, DMSO and aqueous buffer (see Fig. S30, S31 and Table S1). The spectra for [Pt(L^1^)] and [Pt(L^2^)] are very similar, with intense absorption bands below 390 nm which can be assigned to ligand-centred π–π* transitions. Two weak bands can be seen in the visible region (above 410 nm, see Table S1 in the SI) which are due to metal-to-ligand charge transfer (MLCT) transitions. The spectrum of [Pt(L^3^)] is not as well resolved but shows similar features: high intensity bands below 350 nm (due to ligand-centred π–π*) with some additional weak bands in the visible region consistent with the MLCT character (see Table S1 and Fig. S30, S31). The three complexes are emissive in organic solvents, with [Pt(L^1^)] and [Pt(L^2^)] displaying much higher emission intensities in CH_2_Cl_2_ than [Pt(L^3^)]. The latter is significantly red-shifted (*λ*_em_ = 619 nm) as compared to [Pt(L^1^)] (*λ*_em_ = 508 nm) and [Pt(L^2^)] (*λ*_em_ = 520 nm); this is consistent with the emission spectrum of a previously reported non-planar platinum(ii) complex with an analogous tetradentate ligand to L^3^.^39^ The emission of the new complexes is quenched in buffered aqueous medium, which is consistent with the behaviour of other platinum(ii) complexes with cyclometallating and other polypyridyl ligands reported in the literature (although it should be noted that aggregation of this type of complex can lead to red shifted emission; see below for further discussion^32,33,41–43^).

As discussed in the introduction, addition of DNA to platinum(ii) complexes can lead to a ‘switch-on’ effect of their phosphorescence in aqueous media. Therefore, an excess DNA – calf thymus (ct-DNA) as a representative of duplex, and *c-myc* as a representative of quadruplex – was added to aqueous solutions of the three complexes. The emission intensity of [Pt(L^1^)] and [Pt(L^2^)] was clearly switched-on upon DNA addition, while the effect was negligible for [Pt(L^3^)]. This suggests that the square planar complexes bind tightly to DNA preventing quenching processes, while the non-planar [Pt(L^3^)] complex is likely to have a lower affinity for DNA and a different binding mode. Consequently, is expected to be more exposed to the quenching mechanisms, leading to the observed lack of a pronounced emission switch-on effect (see below for further discussion on the origin of the quenching process).

**Fig. 2:**
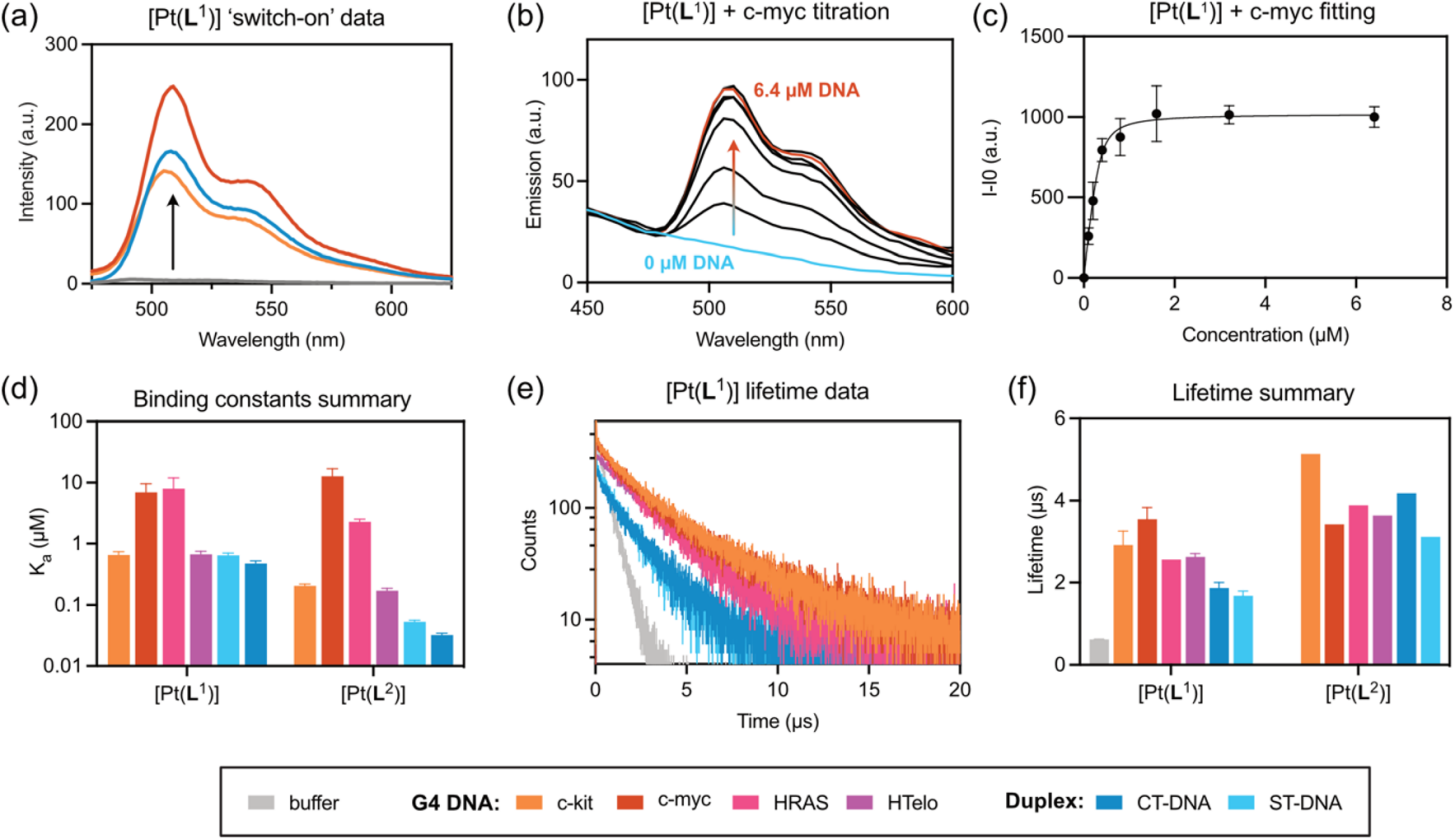
Interactions of [Pt(L^1^)] and [Pt(L^2^)] with DNA: (a) switch-on data for [Pt(L^1^)] with duplex and G4 DNA structures; (b) example of titration data for [Pt(L^1^)] and *c-myc* DNA (excitation at 380 nm); for all other titrations' spectral data see SI; (c) example of a binding curve for [Pt(L^1^)] and *c-myc* DNA (emission monitored at 520 nm); for all other binding curves, see SI; (d) graphical summary of all *K*_a_ values for [Pt(L^1^)] and [Pt(L^2^)], with different DNA structures (*y*-axis values are in log scale). (e) Time-resolved phosphorescence decay traces for [Pt(L^1^)] bound to different DNA structures; (f) summary of phosphorescence lifetime data for both [Pt(L^1^)] and [Pt(L^2^)] when bound to different DNA structures measured by TCSPS (where possible, multiple independent lifetime readings were taken and averaged, as indicated by error bars); measurements were recorded with 6-fold excess of G4 DNAs and 50-fold excess of double-stranded DNAs to ensure complete binding of probe. Colour coding for the different DNA structures is shown at the bottom of the figure.

Based on these results, it was decided to explore in more detail (both *in vitro* and in cells) only the complexes that displayed a clear emission switch-on response: [Pt(L^1^)] and [Pt(L^2^)]. To quantify their DNA binding properties, these two complexes were titrated with increasing amounts of different DNA sequences/topologies (*c-myc*, *c-kit*, *HRAS* and *HTelo* for G4, and ct-DNA and st-DNA for duplex) in aqueous buffer, and their emission intensity recorded (see SI for details). From these titrations, it was possible to determine the *K*_a_ values of the complexes to the different DNA topologies, which are summarised in Fig. 2d, S37–S40 and Table S5. Both complexes display selectivity towards *c-myc* and *HRAS*, which fold into parallel G4 DNA structures. Binding affinity to the other two G4 structures under study (*i.e. c-kit* and *HTelo*) was lower and, in the case of [Pt(L^1^)], was of the same magnitude as the interaction with duplex DNA.

It was also of interest to establish the possible binding modes for these two complexes with G4 and duplex DNA. To assess this, the structures of the three complexes were first optimised using density functional theory (DFT) in Gaussian. As expected, [Pt(L^1^)] and [Pt(L^2^)] have a completely planar structure, while for [Pt(L^3^)] the aromatic rings of the ligand are not coplanar and therefore the overall geometry of the complex is not planar (see Fig. S42). These optimised structures were then used for molecular docking calculations using the experimentally reported structures of *c-myc* DNA (as a representative example of a G4 structure) and an 8-mer B-DNA (as a representative of a duplex structure). As can be seen in Fig. S42, [Pt(L^1^)] and [Pt(L^2^)] interact with G4 DNA *via* end-stacking, while the most favourable interaction with duplex DNA is *via* intercalation. These binding modes are consistent with the values of the affinity constants presented in Fig. 2. In the case of [Pt(L^3^)], the docking calculations showed that it can still bind to the external tetrad of *c-myc* G4 DNA. However, the positioning of the molecule – due to its non-planar nature – does not allow for optimal π–π stacking with the guanine tetrad as compared to the other two planar complexes (particularly [Pt(L^1^)]). With duplex DNA, there is partial intercalation *via* one of [Pt(L^3^)]'s aromatic rings, but with most of the complex positioned in DNA's groove.

Having established the relative DNA binding affinities for [Pt(L^1^)] and [Pt(L^2^)], we then recorded the phosphorescence lifetimes of the two complexes in aqueous buffer with and without DNA. As expected, based on the switch-on properties of these complexes, the phosphorescence lifetime in the absence of DNA is significantly shorter (*τ*_w_ = 0.62 µs) than when bound to any of the DNA structures under study. More interestingly, for complex [Pt(L^1^)] the phosphorescence lifetimes are consistently longer when bound to G4s (*τ*_w_ = 2.5–3.6 µs), as compared to duplex DNA (*τ*_w_ = 1.5–2.0 µs). In the case of [Pt(L^2^)], the increase in lifetime is even larger when bound to DNA (highest *τ*_w_ = 5.1 µs for *c-kit* G4 DNA), but the differences in phosphorescence lifetime between G4s and duplexes are not distinct enough to consistently differentiate between the two topologies.

Having established that [Pt(L^1^)] displays distinct long lifetimes when bound to G4 DNA over duplex DNA and when free in aqueous solution, further studies were performed to gain insights into the possible origin of these observations. As has been previously reported for other square planar platinum(ii) complexes, dioxygen quenches the phosphorescence of this type of complex due to triplet state deactivation.^42^ Thus, the emission lifetimes of an aqueous solution of [Pt(L^1^)] under normal aerated conditions and after bubbling with N_2_ were recorded (see Table S3). The lifetime of the probe in water is 0.61 µs, but after bubbling N_2_ there is a large increase in the lifetime to 6.77 µs. This confirms the expected quenching role of oxygen when the probe is in solution. Next, we recorded the emission lifetime of mixtures of [Pt(L^1^)] with G4 (*c-myc* and *c-kit*) and duplex DNA (st DNA) under ambient conditions and after bubbling N_2_. As can be seen in Table S3, the lifetime is significantly longer when the probe is bound to DNA under both conditions. However, the solution deaerated by bubbling N_2_, shows emission lifetimes comparable to that of deaerated water. These experiments suggest that upon binding to different topologies of DNA, the ability of dioxygen to quench the emission of the probe is significantly reduced. The local environment around the probe (*i.e.* whether it binds to duplex or quadruplex DNA) seems to play a very important role in restricting the ability of oxygen to quench the emission. Therefore, the lifetime of [Pt(L^1^)] is highly dependent on the topology it binds to, with consistently longer emission lifetimes for G4s *vs.* duplex DNA structures.

### Confocal microscopy and PLIM

**Fig. 3:**
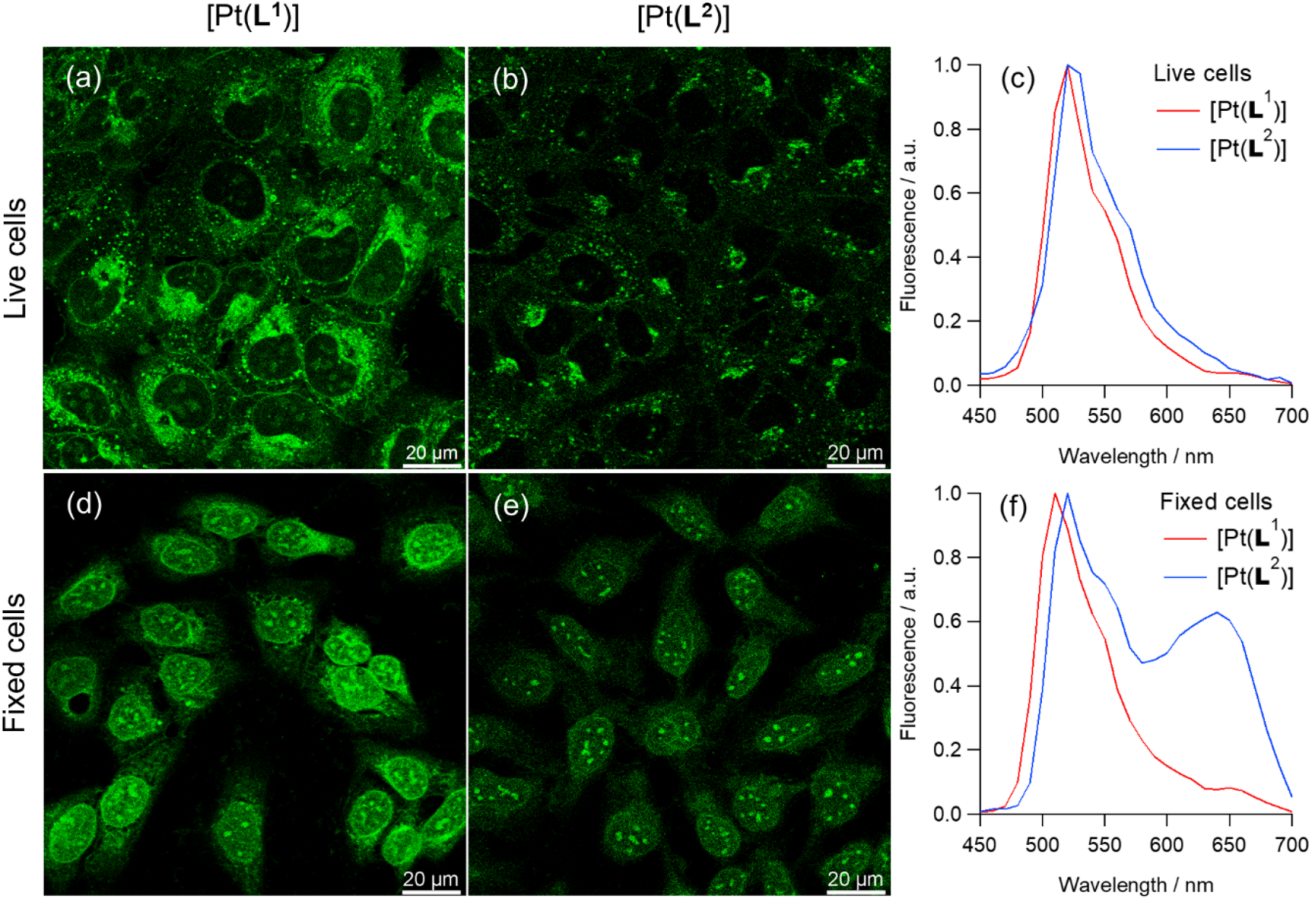
Confocal images recorded for (a and b) live and (d and e) fixed U2OS cells stained with 5 µM of (a and d) [Pt(L^1^)] and (b and e) [Pt(L^2^)] 7 min after the addition of the corresponding probe into the media. Images recorded following 780 nm multiphoton excitation and detection in the 480–580 nm region for [Pt(L^1^)] and [Pt(L^2^)]. Scale bars are 20 µm. (c and f) Emission spectra recorded with live and fixed U2OS cells.

In live cells, both [Pt(L^1^)] and [Pt(L^2^)] are primarily located in cytosolic organelles with modest penetration into the nucleus, as visualised by low-intensity signals from nucleoli (Fig. 3). The staining pattern changes over extended incubation time, with most of the fluorescence after 3 h incubation originating from granular-like structures (Fig. S43 and S44). In contrast, in fixed cells, most of the fluorescence originates from the nucleus, with particularly bright staining of nucleoli, for both complexes (Fig. 3). By recording emission spectra of [Pt(L^1^)] and [Pt(L^2^)] from live and fixed cells, we detected two emission bands: centred at 530 nm and 660 nm, with various intensities in different organelles. The intensity of the bands was also dependent on the incubation conditions, with longer incubation times promoting the appearance of the 660 nm band, particularly in lysosomes. As mentioned above, square planar platinum(ii) complexes can aggregate in aqueous solution leading to red-shifted MMLCT-type emission bands.^43^ To study whether the 660 nm band observed in cells was indeed caused by probe aggregation, we recorded the emission spectra of an aqueous solution of [Pt(L^1^)] at various concentrations. As can be seen in Fig. S34, up to 17 µM, only the band at 530 nm is observed (*i.e.* the non-aggregated probe). However, at 30 µM a new band at 680 nm appears. Interestingly, in aqueous solution, [Pt(L^2^)] starts to aggregate at 2 µM. Therefore, the 660 nm emission band, seen under certain conditions in the cellular-derived spectra for these two complexes, particularly for [Pt(L^2^)], was assigned to aggregated species, by similarities with earlier studies^34,41^ and consistent with the fact that it was seen when cells were treated with higher concentrations of complex.

Considering the above, dual colour phosphorescence imaging was performed for [Pt(L^1^)] and [Pt(L^2^)] (Fig. S43–S45), in live and fixed cells, detecting monomer emission between 480–580 nm and aggregate emission between 610–690 nm. These studies, as well as emission spectra (Fig. 3c and f), demonstrated that no aggregate emission was detected for [Pt(L^1^)] at short incubation times, while imaging under various incubation conditions and fixation methods, resulted in significant redshifted emission for [Pt(L^2^)] associated with aggregation of the complex. This observation, combined with the fact that [Pt(L^2^)] does not display selective phosphorescence lifetime for G4 DNA, makes this complex unsuitable for imaging quadruplexes in cells and therefore it was not studied any further.

While some red emission centred at 660 nm, assigned to aggregated species, was detected for [Pt(L^1^)] at longer incubation times in live cells (Fig. S43), green *vs.* red channel co-localisation imaging experiments clearly demonstrated that the red emission at 660 nm originates exclusively from granule-like structures and shows no overlap with regions displaying the green emission (monomer).

**Fig. 4:**
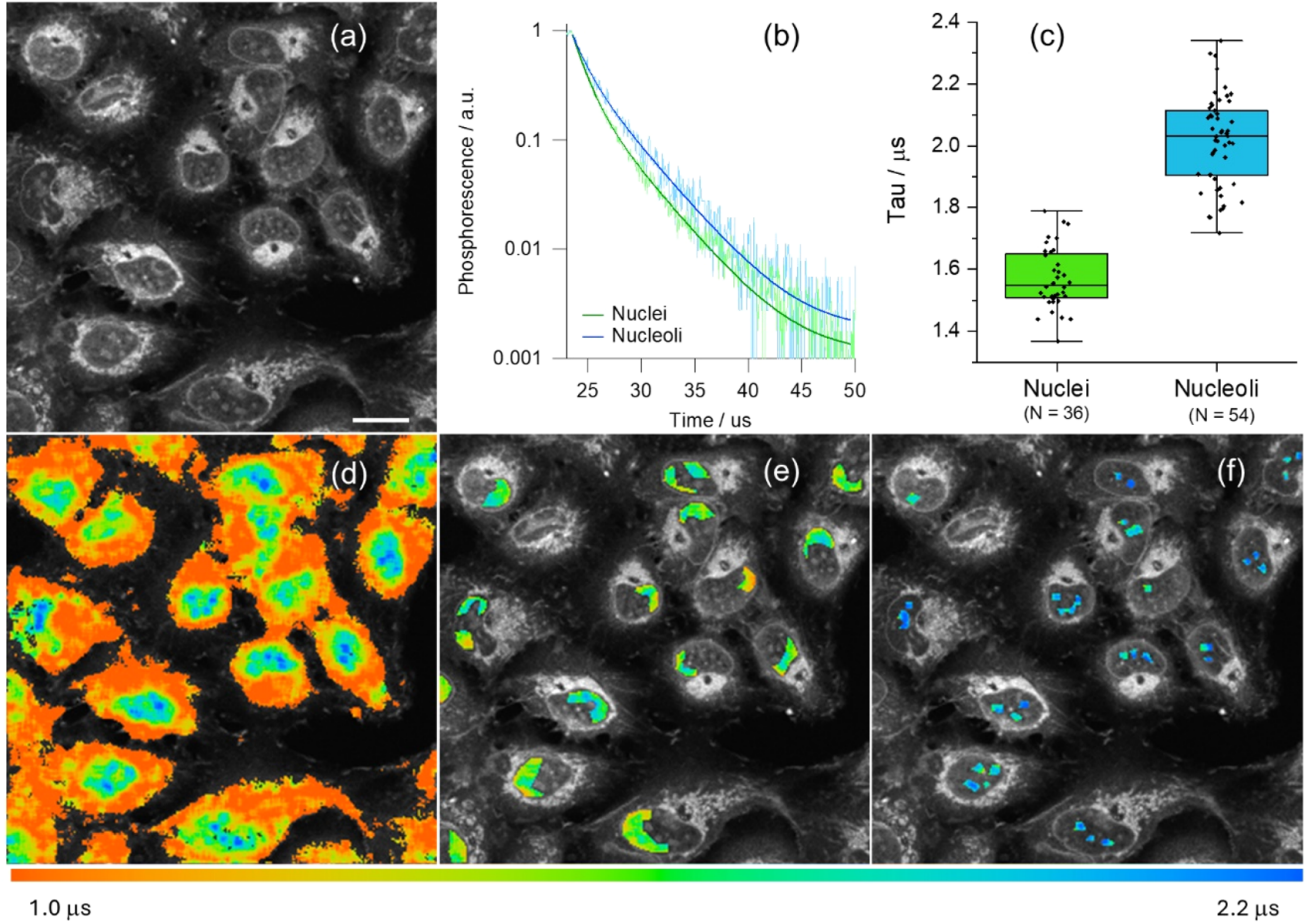
Optical imaging of live U2OS cells incubated with [Pt(L^1^)]. (a) Intensity image; (b) normalised averaged phosphorescence decay traces corresponding to segments in (e) and (f); (c) associated statistical cell-by-cell ROI analysis, *p* < 0.05 for all condition pairs; (d) unsegmented PLIM images of live U2OS cells stained with 5 µM of [Pt(L^1^)] for 15 min (780 nm multiphoton excitation and detection in the 480–580 nm region; scale bar is 20 µm); (e) segmented PLIM images of nuclei excluding nucleoli; (f) segmented PLIM images of nucleoli.

PLIM datasets were recorded for [Pt(L^1^)] at early stages of incubation in live U2OS cells, at conditions where no aggregation-assigned red emission was seen. [Pt(L^1^)] showed sufficient signal intensity from nuclei for reliable analysis of phosphorescence lifetimes (Fig. 4). Unsegmented PLIM images of live U2OS cells incubated with [Pt(L^1^)] (Fig. 4d) demonstrate a clear correlation of lifetime values with different organelles: the shortest lifetimes seen within cytosolic organelles (*ca*. 1.2 µs), longer lifetimes in nuclei (*ca*. 1.6 µs) and the longest values seen in nucleoli (*ca*. 2.1 µs). Significant differences in lifetimes within nuclei were confirmed by segmentation of corresponding areas, with averaged kinetic curves as well as individual region of interest (ROI)-wise value distributions (Fig. 4b, e and 4f, respectively). While the [Pt(L^1^)] lifetimes within nuclei are of the same magnitude than those recorded for the probe with ct-DNAs *in vitro* (see Table S6), the longer lifetimes in the nucleoli may indicate a contribution from [Pt(L^1^)] interacting with G4 quadruplexes, which are known to be present in higher concentrations within nucleoli.^44^ Likewise, long phosphorescence lifetimes were detected for [Pt(L^1^)] in fixed cells, Fig. S46. Overall, much brighter signals were detected from fixed cells, with both the nuclear lifetime values (*ca.* 2.6 µs) and nucleolar lifetimes (*ca.* 2.75 µs) falling in the ‘quadruplex’ region of lifetimes of [Pt(L^1^)] (*τ*_w_ = 2.5–3.6 µs, Table S6). It is noteworthy, that signal intensities were higher and lifetimes of [Pt(L^1^)] were longer in fixed cells compared to live cells, which may indicate higher accessibility of G4s upon cell fixation.

**Fig. 5:**
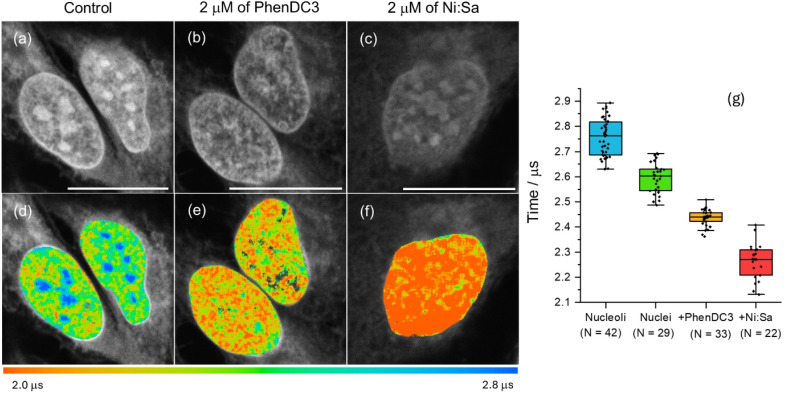
(a–c) Phosphorescence intensity and (d–f) lifetime (PLIM) images of fixed U2OS cells stained with 5 µM of [Pt(L^1^)] (780 nm multiphoton excitation and detection in the 480–580 nm region; scale bars are 20 µm). Images for the sample treated with (b and e) PhenDC3 (2 mM) and (c and f) Ni-Salphen (2 mM) for 1 h prior to fixation. (g) Statistical analysis of individual ROIs phosphorescence lifetimes, recorded in control cells and in the presence of competitive binders (*p* < 0.05 for all condition pairs).

To confirm whether the longer phosphorescence lifetime in nucleus/nucleoli originated from the interaction of [Pt(L^1^)] with G4s, PLIM data was recorded in fixed cells pre-stained with well-known G4 binders, namely PhenDC3 ^45^ and Ni-Salphen.^46^ In the presence of these ligands, [Pt(L^1^)] does not stain nucleoli, exhibiting weaker signal intensities (Fig. 5a–c and S47) and shorter lifetimes (Fig. 5d–f). This data is consistent with the two G4 binders blocking [Pt(L^1^)] from accessing G4 DNA binding sites, with a higher effect displayed by Ni-Salphen.^24,34^

## Conclusions

Three new platinum(ii) complexes coordinated to C^N^N^C or N^C^C^N cyclometallating ligands have been successfully synthesised. Our initial hypothesis, namely that these complexes would bind selectively to G4s and work as phosphorescent probes by switching-on their emission upon DNA binding, was confirmed for [Pt(L^1^)] and [Pt(L^2^)]. In the case of [Pt(L^3^)] – which has a non-planar geometry – its interaction with DNA does not lead to enhanced phosphorescence, and therefore it was not taken forward for further studies. [Pt(L^1^)] showed to have distinctly longer phosphorescence lifetime when bound to G4 DNA, as compared to when it is bound to duplex DNA or free in solution; while [Pt(L^2^)] did not show a consistent lengthening of its phosphorescence decay when bound to G4 structures. The emission intensity switch-on and lengthening of the lifetime observed when [Pt(L^1^)] and [Pt(L^2^)] interact with DNA, seem to be due to changes in the ability of oxygen to quench the emission of the complexes. In the case of [Pt(L^1^)], deactivation of the quenching mechanism is particularly sensitive to the DNA topology the probe is bound to, making this a very selective probe to distinguish G4 *vs.* duplex DNA. Both these probes were studied by microscopy in live and fixed cells, which showed that [Pt(L^1^)] stains the nucleus (and nucleoli) better than [Pt(L^2^)]. Importantly, it was observed that [Pt(L^2^)] aggregates when localised in cellular compartments – leading to a redshifted emission – making it unsuitable for G4 imaging. In contrast, [Pt(L^1^)] showed to be a good probe to image G4s by PLIM, as demonstrated by the longer lifetimes observed in nucleoli and the shortening of lifetimes when strong G4 binders were incubated with cells as competitive G4 binders. Altogether, the *in vitro* and cellular data shows [Pt(L^1^)] to be an excellent probe to visualise G4 DNA in cells, overcoming the problem of probe aggregation that was previously encountered with phosphorescent PLIM-based G4 probes.

## Author contributions

AB, PSS and TK performed the experiments. All authors contributed to analysis of the experimental data. MKK and RV designed and supervised the project. All authors contributed to writing the manuscript and approved the final version.

## Conflicts of interest

There are no conflicts to declare.

## Supplementary Material

SC-017-D5SC08064A-s001

## Data Availability

The data that support the findings of herein reported are available within the article and the supplementary information (SI). Supplementary information: materials and methods, experimental procedures, characterisation data, and NMR spectra, mass spectra, LC-MS data, fluorescence titrations, UV/vis and emission spectra, and SI microscopy figures. See DOI: https://doi.org/10.1039/d5sc08064a.
