# Supplementary material for: Platinum-based phosphorescent lifetime probes for the visualisation of G-quadruplex DNA in cells
Source: Chem Sci. 2026 Feb 4;17(14):7018–26. doi: 10.1039/d5sc08064a (PMC12898780; doi:10.1039/d5sc08064a)
Supplement: SC-017-D5SC08064A-s001 [file SC-017-D5SC08064A-s001.pdf]

Supplementary Information (SI) for Chemical Science.
This journal is © The Royal Society of Chemistry 2026

Platinum-based phosphorescent lifetime probes for the visualisation of G-quadruplex DNA in cells

Supplementary Information

Experimental Details

General Procedures. All starting materials were purchased from Sigma–Aldrich, Fluorochem or VWR and used as received from commercial sources. ^{1}H NMR and ^{13}C NMR spectra were recorded on a Bruker Avance 400 MHz or 500 MHz Ultrashield NMR spectrometer and chemical shifts are reported in parts per million (ppm). Mass spectrometric analysis was performed on an Agilent 6125 MS/1260 LC. Absorbance measurements were done using an 8453 UV/Visible Spectroscopy System (Agilent) or a Cary 60 UV/Vis Spectrometer (Agilent) and a 1 cm quartz cuvette. All fluorescence measurements were measured by using a Fluoromax-4 spectrofluorometer (Jobin–Yvon; Horiba) using a 1 cm quartz cuvette or BMG Clariostar Microplate reader (see details in the specific sections). Compounds **1**, **2** and **4** were synthesized and characterized according to published procedures.[1]

Synthesis of 3.

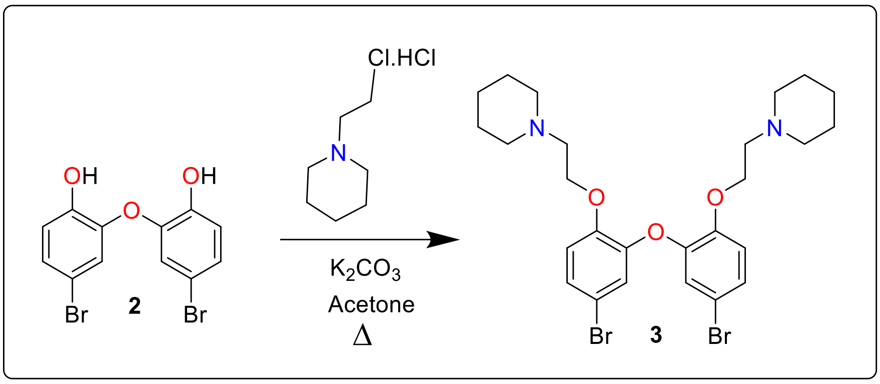

An oven dried 250 mL two-neck flask was charged with compound **2**[1] (500 mg, 1.39 mmol), 1-(2-chloroethyl)piperidine hydrochloride (766 mg, 4.17 mmol) and K_2CO_3 (959 g, 6.95 mmol). To this anhydrous acetone (80 mL) was added, and the resulting mixture was heated at 60 °C for 48 hours under N_2. After cooling to room temperature, the solid residue was removed by filtration and washed with dichloromethane and the filtrate was concentrated by rotatory evaporation. The crude product was extracted with CH_2Cl_2 and dried over Na_2SO_4, and concentrated under reduced pressure. The resulting crude product was purified by column chromatography on a neutral alumina, using EtOAc as the eluent to afford compound **3** as a white solid. Yield 68%. ^{1}H NMR (400 MHz, $CDCl_3$) δ 7.17 (d, J = 8.5 Hz, 2H), 6.95 (s, 2H), 6.85 (d, J = 8.7 Hz, 2H), 4.17 (d, J = 6.6 Hz, 4H), 2.77 (s, 4H), 2.49 (s, 8H), 1.68 – 1.49 (m, 8H), 1.42 (t, J = 6.5 Hz, 4H). ^{13}C NMR (101 MHz, $CDCl_3$) δ 148.79, 146.68, 127.25, 122.51, 115.92, 113.09, 67.31, 57.57, 54.94, 25.65, 23.90. TOF MS (ES^+) calculated for $C_{26}H_{35}N_2O_3$ = 583.0994, found = 583.0993.

Synthesis of 5.

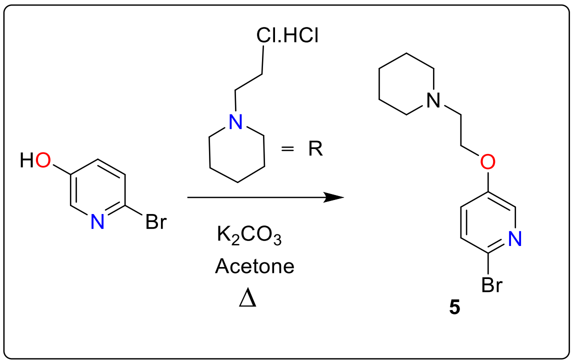

5

In an oven dried 250 ml RB was charged with a mixture of 2-Bromo-5-hydroxypyridine (1.52 g, 8.74 mmol), 1-(2-Chloroethyl)piperidine hydrochloride (2.40 g, 13.04 mmol) K_2CO_3(3.99 g, 28.87 mmol). Acetone (80 mL) was added and allowed to stir at 60 °C for 72 h under N_2. After completion of the reaction, the solid component was removed by filtration and washed with DCM and the filtrate was concentrated by rotatory evaporation. The residue was then purified by column chromatography on silica-gel using neutral alumina (100 % ethyl acetate) to afford compound **5** as a pale-yellow oil. Yield 81.5%. ^{1}H NMR (400 MHz, $CDCl_3$) δ 8.05 (d, J = 3.1 Hz, 1H), 7.34 (d, J = 8.7 Hz, 1H), 7.10 (dd, J = 8.7, 3.2 Hz, 1H), 4.10 (t, J = 5.9 Hz, 2H), 2.75 (t, J = 5.9 Hz, 2H), 2.48 (t, J = 5.4 Hz, 4H), 1.58 (q, J = 5.7 Hz, 4H), 1.43 (t, J = 6.0 Hz, 2H). ^{13}C NMR (101 MHz, $CDCl_3$) δ 154.87, 137.68, 132.23, 128.19, 125.13, 66.96, 57.83, 55.22, 25.99, 24.21. TOF MS (ES$^+$) calculated for $C_{12}H_{18}N_2OBr$ = 285.0602, found = 285.0599

Synthesis of 6.

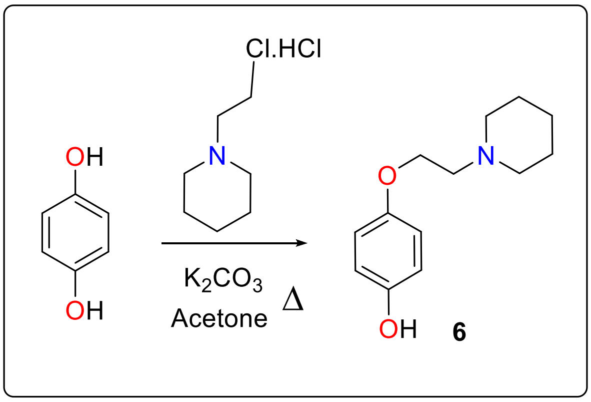

6

A 250 ml round-bottom flask, pre-dried in an oven, was charged with a mixture comprising hydroquinone (1.100 g, 10 mmol), 1-(2-chloroethyl)piperidine (1.656 g, 9 mmol), and K_2CO_3 (4.14 g, 30 mmol). Anhydrous Acetone (80 mL) was added, and the resulting mixture was stirred at room temperature for 72 hours under a nitrogen atmosphere. After completion of the reaction, the solid component was removed by filtration and washed with CH_2Cl_2 and the filtrate was concentrated by rotatory evaporation. The resulting crude mixture was purified by column chromatography on silica gel, utilizing ethyl acetate/2M NH_4OH solution in ethanol (9:1) as an eluent to afford compound **6** as a white solid. Yield 29%. ^{1}H NMR (400 MHz, MeOD) δ 6.79 – 6.74 (m, 2H), 6.72 – 6.67 (m, 2H), 4.91 (s, 2H), 4.03 (t, *J* = 5.7 Hz, 2H), 2.73 (t, *J* = 5.7 Hz, 2H), 2.54 (t, *J* = 5.6 Hz, 4H), 1.62 (q, *J* = 5.7 Hz, 4H), 1.52 – 1.44 (m, 2H). ^{13}C NMR (101 MHz, MeOD) δ 153.46,

152.48, 116.80, 116.59, 67.03, 59.14, 55.92, 26.43, 25.03. TOF MS (ES$^+$) calculated for $C_{13}H_{20}NO_2$ = 222.1494, found = 222.1483.

Synthesis of L^1.

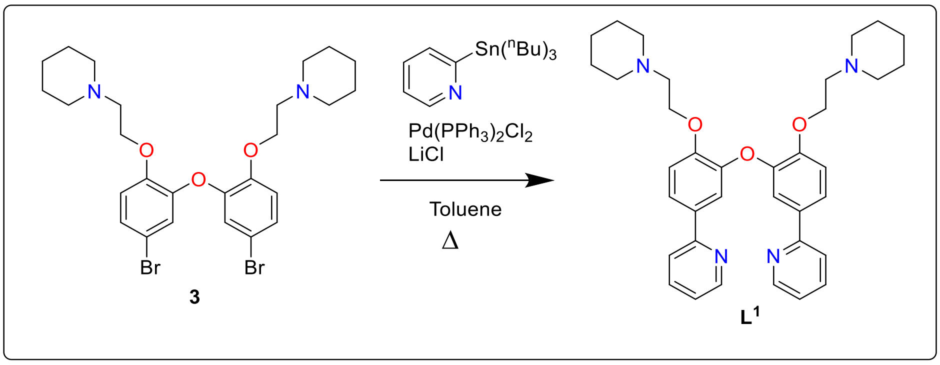

The solvent was degassed using three freeze-pump-thaw cycles prior to the reaction. An oven-dried Schlenk tube was charged with a mixture of compound **3** (290 mg, 0.5 mmol), 2-(tri-n-butylstannyl)pyridine (0.640 mL, 2.0 mmol), $[Pd(PPh_3)_2Cl_2]$ (35 mg, 0.05 mmol), and LiCl (172 mg, 4 mmol) in toluene (6 mL). The reaction mixture was stirred under reflux for 2 days under a nitrogen atmosphere. After cooling to room temperature, the reaction mixture was filtered, and the residue was washed with CH_2Cl_2. The combined filtrate was poured into water, extracted with CH_2Cl_2, and dried over anhydrous Na_2SO_4. The resulting solution was concentrated under reduced pressure. The resulting crude product was purified by column chromatography on a silica gel column, using a mixture of EtOAc/MeOH (10:1) as the eluent, to afford a white solid identified as compound $\mathbf{L^1}$. Yield 58%. ^{1}H NMR (400 MHz, $CDCl_3$) δ 8.58 (ddd, J = 4.9, 1.9, 0.9 Hz, 2H), 7.75 (dd, J = 8.5, 2.2 Hz, 2H), 7.64 (td, J = 7.7, 1.8 Hz, 2H), 7.54 (dd, J = 6.8, 1.7 Hz, 4H), 7.12 (ddd, J = 7.4, 4.8, 1.1 Hz, 2H), 7.08 (d, J = 8.5 Hz, 2H), 4.22 (t, J = 6.1 Hz, 4H), 2.72 (t, J = 6.1 Hz, 4H), 2.41 (t, J = 5.4 Hz, 8H), 1.50 (q, J = 5.6 Hz, 8H), 1.36 (t, J = 6.2 Hz, 4H). ^{13}C NMR (101 MHz, $CDCl_3$) δ 156.71, 150.70, 149.66, 146.68, 136.76, 132.90, 122.67, 121.72, 120.05, 117.92, 114.50, 67.50, 57.87, 55.10, 26.07, 24.26. TOF MS (ES$^+$) calculated for $C_{36}H_{43}N_4O_3$ = 579.3335, found = 579.3315.

Synthesis of L^2.

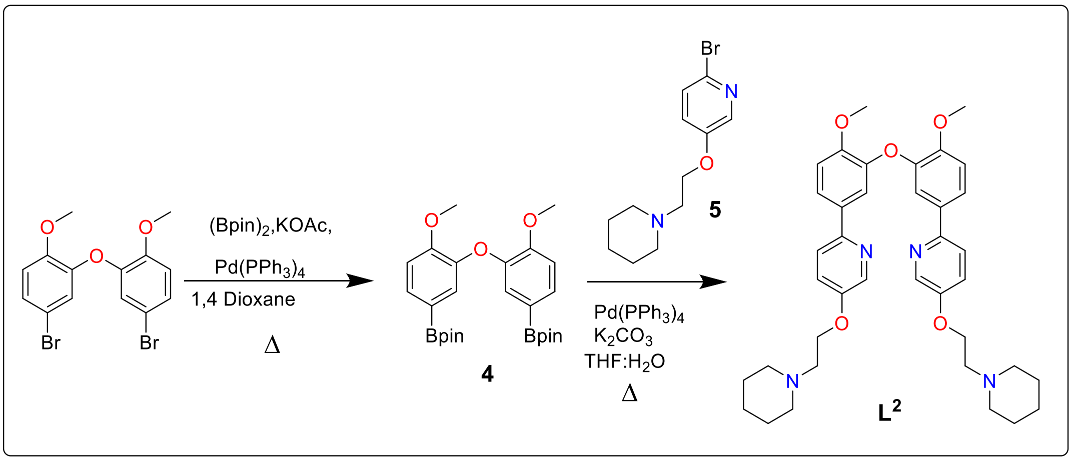

The solvent mixture THF: Water (4:1) was purged with N_2 gas prior to the reaction. A dry Schlenk tube was charged with a mixture of **4**[1] (200 mg, 0.415 mmol), **5** (354 g, 1.245 mmol), K_2CO_3 (344

mg, 2.49 mmol) and [Pd(PPh_3)$_4$] (47 mg, 0.042 mmol). To the reaction vessel the solvent THF: Water (4:1) (15 mL) mixture was added and allowed to stir at 70 °C overnight under inert atmosphere. The reaction mixture was filtered and washed with ethyl acetate and the solvent was removed under reduced pressure. The crude mixture was extracted with ethyl acetate and dried over Na_2SO_4. The solution was concentrated by rotary-evaporator to dryness. The crude product mixture was then flash chromatographed on a silica-gel column using a mixture of CH_2Cl_2/2M NH_4OH in EtOH (10:1) to afford a white solid identified as ligand **L^2**. Yield 54%. ^{1}H NMR (400 MHz, $CDCl_3$) δ 8.27 (d, *J* = 2.9 Hz, 2H), 7.70 (dd, *J* = 8.5, 2.2 Hz, 2H), 7.48 – 7.41 (m, 4H), 7.17 (dd, *J* = 8.8, 3.0 Hz, 2H), 7.05 (d, *J* = 8.6 Hz, 2H), 4.14 (t, *J* = 6.0 Hz, 4H), 3.90 (s, 6H), 2.77 (t, *J* = 5.9 Hz, 4H), 2.58 – 2.45 (m, 8H), 1.60 (q, *J* = 5.6 Hz, 8H), 1.44 (q, *J* = 6.2 Hz, 4H). ^{13}C NMR (101 MHz, $CDCl_3$) δ 153.86, 150.92, 149.57, 146.14, 137.50, 132.49, 122.22, 122.08, 120.34, 117.01, 112.71, 66.57, 57.94, 56.25, 55.20, 25.98, 24.24. TOF MS (ES$^+$) calculated for $C_{38}H_{47}N_4O_5$ = 639.3546, found = 639.3536.

Synthesis of L^3.

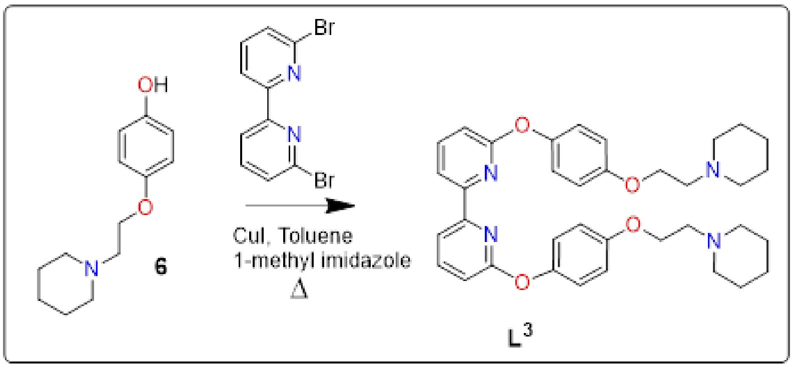

Under a nitrogen atmosphere, a dry Schlenk tube was charged with 6,6'-dibromo-2,2'-bipyridine (200 mg, 0.64 mmol), **6** (352 mg, 1.59 mmol), 1-methylimidazole (0.051 ml, 0.64 mmol), K_2CO_3 (353 g, 2.56 mmol), and anhydrous toluene (10 mL). The solution was degassed by the method of freeze– pump–thaw cycles for three cycles. Copper(I) iodide (24 mg, 0.128 mmol) was then added, and the solution was degassed by the method mentioned above for another 3 times, and the vessel was sealed. The mixture was stirred and heated at 110 °C under a nitrogen atmosphere for 3 days. Upon cooling to room temperature, the mixture was filtered and washed with CH_2Cl_2. The filtrate was then poured into water and was extracted with CH_2Cl_2. The organic phase was separated and dried over anhydrous Na_2SO_4 and concentrated under reduced pressure. The residue was then purified by column chromatography on silica using EtOAc/MeOH (9:1) as an eluent to gain **L^3** as a white solid. Yield 60%. ^{1}H NMR (400 MHz, $CDCl_3$) δ 7.85 (dd, *J* = 7.6, 0.8 Hz, 1H), 7.71 – 7.64 (m, 1H), 7.14 – 7.09 (m, 2H), 6.97 – 6.91 (m, 2H), 6.77 (dd, *J* = 8.2, 0.8 Hz, 1H), 4.14 (t, *J* = 6.0 Hz, 2H), 2.82 (t, *J* = 6.0 Hz, 2H), 2.55 (s, 4H), 1.64 (p, *J* = 5.6 Hz, 4H), 1.51 – 1.43 (m, 2H). ^{13}C NMR (101 MHz, $CDCl_3$) δ 163.50, 155.42, 153.71, 147.95, 140.38, 122.59, 115.55, 115.46, 110.93, 77.40, 65.73, 57.79, 55.03, 25.29, 23.76. TOF MS (ES$^+$) calculated for $C_{36}H_{43}N_4O_4$ = 595.3284, found = 595.3294.

Synthesis of [Pt(L[1])].

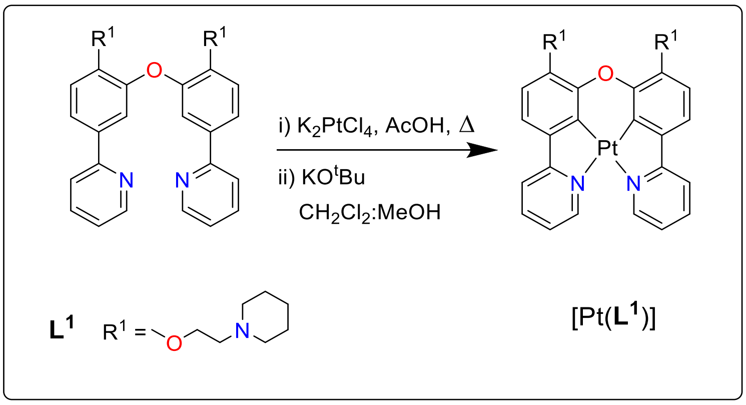

The solvent was degassed by performing three freeze-pump-thaw cycles prior to the reaction. Under a nitrogen atmosphere, a dry Schlenk tube was charged with compound $\mathbf{L^1}$ (40 mg, 0.069 mmol) and K_2PtCl_4 (35 mg, 0.083 mmol). Degassed glacial acetic acid (6 mL) was then added, and the resulting mixture was heated at 100 °C for 72 hours. After cooling to room temperature, the crude reaction mixture was poured into water. The resulting yellow to orange precipitate was collected by filtration, washed with water and diethyl ether, and dried in air. The crude yellow solid was used directly in the next step without further purification. In the second step, a dry Schlenk tube was charged with the crude product and treated with excess KOtBu (20 equivalents). A degassed, anhydrous solvent mixture of CH_2Cl_2:MeOH (1:2) was added, and the resulting mixture was stirred at room temperature for 5 hours. Upon completion of the reaction, the mixture was filtered, and the filtrate was concentrated by rotary evaporation. The resulting yellow solid was dissolved in CH_2Cl_2 and passed through a neutral alumina column. Pure fractions were collected, concentrated by rotary evaporation, washed with diethyl ether, and dried to yield [Pt($\mathbf{L^1}$)] as a yellow solid. Yield 28%. ^1H NMR (400 MHz, CD_2Cl_2) δ 8.93 – 8.86 (m, 2H), 7.95 – 7.88 (m, 4H), 7.53 (d, J = 8.2 Hz, 2H), 7.36 (td, J = 5.4, 3.4 Hz, 2H), 6.91 (d, J = 8.2 Hz, 2H), 4.40 (s, 4H), 2.99 (s, 4H), 2.70 (s, 8H), 1.73 – 1.63 (m, 8H), 1.50 (d, J = 7.4 Hz, 4H). ^{13}C NMR (126 MHz, CD_2Cl_2) δ 165.78, 148.14, 141.16, 139.58, 138.60, 127.57, 125.25, 121.73, 119.68, 119.54, 109.31, 66.91, 58.37, 26.05, 24.41. ^{195}Pt NMR (500 MHz, CD_2Cl_2) = -3713.2849. TOF MS (ES^+) calculated for $C_{36}H_{41}N_4O_3Pt$ = 772.2827, found = 772.2838. Elemental analysis calcd for $C_{36}H_{40}N_4O_3Pt{\cdot}1.75CH_2Cl_2$: C 49.26, H 4.76, N 6.09; found: C 49.29, H 4.59, N 5.90.

Synthesis of [Pt(L^2)].

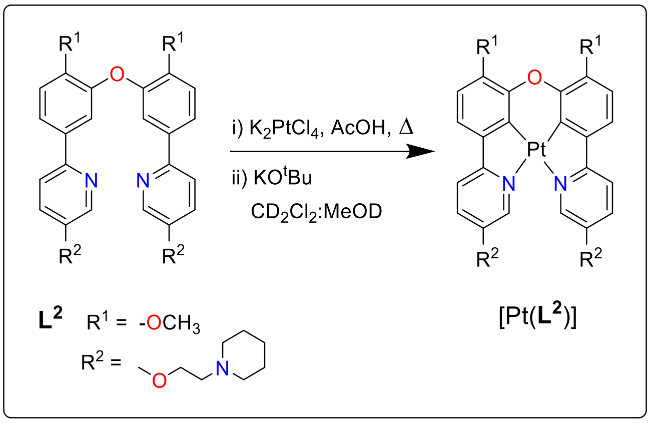

The solvent was degassed by performing three freeze-pump-thaw cycles prior to the reaction. Under a nitrogen atmosphere, a dry Schlenk tube was charged with compound **L^2** (40 mg, 0.063 mmol) and K_2PtCl_4 (32 mg, 0.075 mmol). Degassed glacial acetic acid (6 mL) was then added, and the resulting mixture was heated at 100 °C for 72 hours. After cooling to room temperature, the crude reaction mixture was poured into water. The resulting yellow to orange precipitate was collected by filtration, washed with water and diethyl ether, and dried in air. The crude yellow solid was used directly in the next step without further purification. In the second step, a dry Schlenk tube was charged with the crude product and treated with excess KOtBu (20 equivalents). A degassed, anhydrous solvent mixture of CH_2Cl_2:MeOH (1:2) was added, and the resulting mixture was stirred at room temperature for 5 hours. Upon completion of the reaction, the mixture was filtered, and the filtrate was concentrated by rotary evaporation. The resulting yellow solid was dissolved in CH_2Cl_2 and passed through a neutral alumina column. Pure fractions were collected, concentrated by rotary evaporation, washed with diethyl ether, and dried to yield [Pt(**L^2**)] as a yellow solid. Yield 23%. ^{1}H NMR (400 MHz, CD_2Cl_2) δ 8.57 (d, J = 2.8 Hz, 2H), 7.83 (d, J = 9.0 Hz, 2H), 7.52 (dd, J = 8.9, 2.7 Hz, 2H), 7.41 (d, J = 8.2 Hz, 2H), 6.87 (d, J = 8.2 Hz, 2H), 4.26 (s, 4H), 4.03 (s, 6H), 2.84 (s, 4H), 2.55 (s, 8H), 1.63 – 1.60 (m, 8H), 1.49 – 1.44 (m, 4H). ^{13}C NMR (126 MHz, CD_2Cl_2) δ 154.21, 150.15, 140.87, 139.27, 136.50, 125.43, 124.30, 119.75, 118.42, 107.20, 67.55, 58.18, 56.37, 55.44, 26.33, 24.53. ^{195}Pt NMR (500 MHz, CD_2Cl_2) = -3689.8686.

Synthesis of [Pt(L^3)].

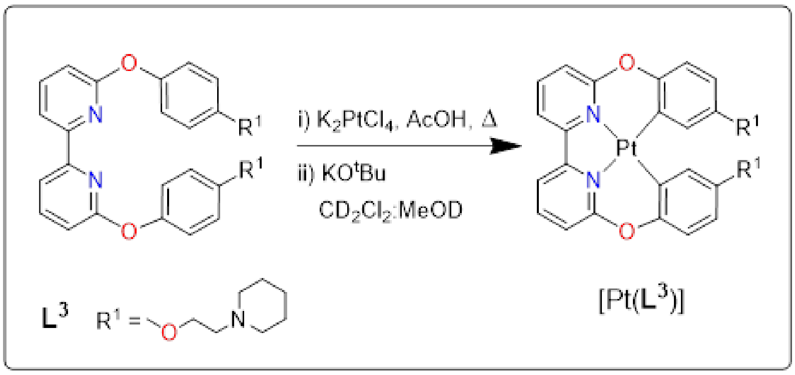

The solvent was degassed by performing three freeze-pump-thaw cycles prior to the reaction. Under a nitrogen atmosphere, a dry Schlenk tube was charged with compound **L^3** (45 mg, 0.076

mmol), K_2PtCl_4 (38 mg, 0.091 mmol). Degassed glacial acetic acid (6 mL) was then added, and the resulting mixture was heated at 100 °C for 72 hours. After cooling to room temperature, the crude reaction mixture was poured into water. The resulting yellow to orange precipitate was collected by filtration, washed with water and diethyl ether, and dried in air. The crude yellow solid was used directly in the next step without further purification. In the second step, a dry Schlenk tube was charged with the crude product and treated with excess KOtBu (20 equivalents). A degassed, anhydrous solvent mixture of CH_2Cl_2:MeOH (1:2) was added, and the resulting mixture was stirred at room temperature for 5 hours. Upon completion of the reaction, the mixture was filtered, and the filtrate was concentrated by rotary evaporation. The resulting yellow solid was dissolved in CH_2Cl_2 and passed through a neutral alumina column. Pure fractions were collected, concentrated by rotary evaporation, washed with diethyl ether, and dried to yield [Pt(**L^3**)] as a yellow solid. Yield 33%. ^{1}H NMR (400 MHz, MeOD) δ 8.21 (t, J = 7.9 Hz, 2H), 8.10 (d, J = 7.6 Hz, 2H), 7.50 (d, J = 8.3 Hz, 2H), 7.34 (d, J = 3.2 Hz, 1H), 7.19 (d, J = 8.8 Hz, 2H), 6.72 (dd, J = 8.7, 3.0 Hz, 2H), 4.17 (t, J = 5.5 Hz, 4H), 3.03 (t, J = 5.6 Hz, 4H), 2.81 (s, 7H), 1.69 (q, J = 5.8 Hz, 8H), 1.55 (d, J = 6.1 Hz, 4H). ^{13}C NMR (126 MHz, CD_2Cl_2) δ 156.89, 154.25, 148.63, 139.36, 127.82, 125.81, 125.25, 117.11, 116.68, 116.50, 111.86, 63.93, 57.08, 54.78, 23.49, 22.41. ^{195}Pt NMR (400 MHz, CD_3OD) = -3272.0630. TOF MS (ES$^+$) calculated for $C_{36}H_{41}N_4O_4Pt$ = 788.2776, found = 788.2813. Elemental analysis calcd for $C_{36}H_{40}N_4O_3Pt\cdot0.45CH_2Cl_2$: C 53.00, H 4.99, N 6.78; found: C 53.13, H 4.96, N 6.74.

Spectroscopic data for compound 3.

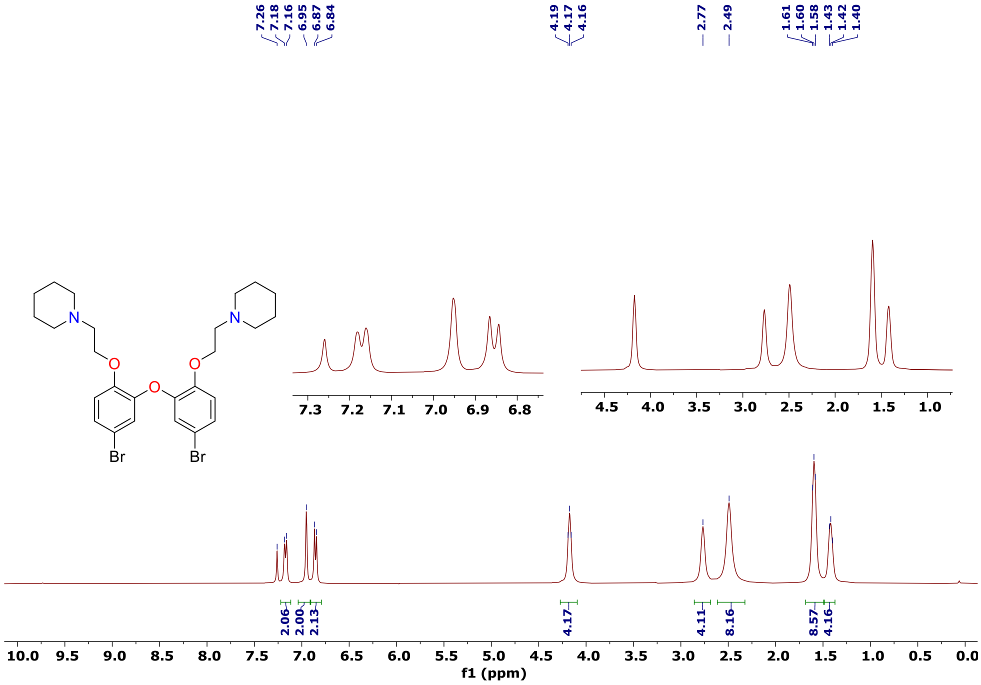

Figure S1. ^{1}H NMR spectrum in $CDCl_3$ of compound **3**

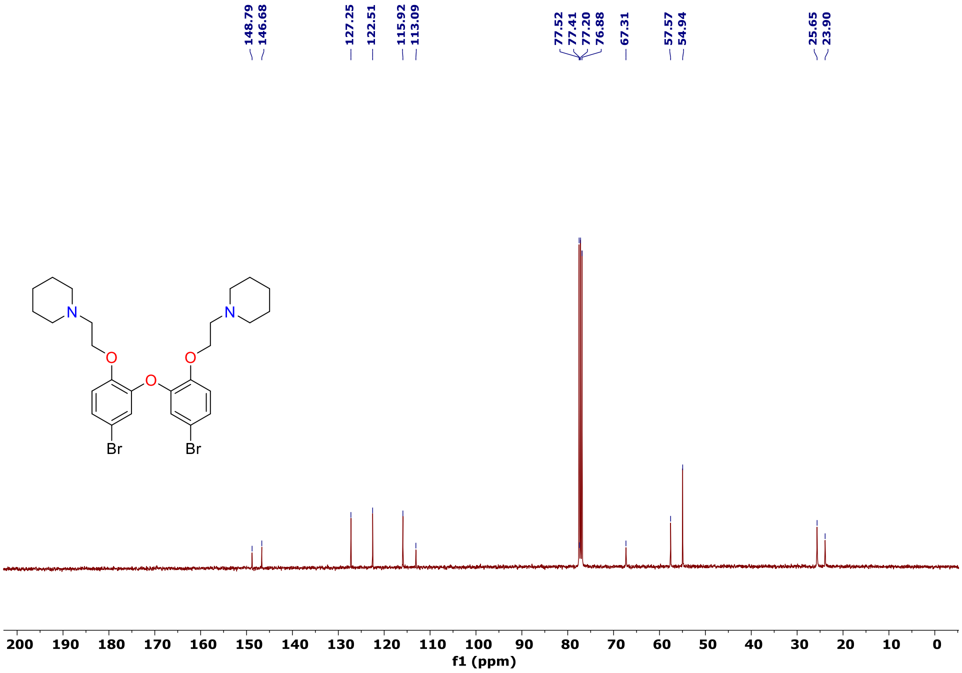

Figure S2. ^{13}C NMR spectrum in $CDCl_3$ of compound **3**

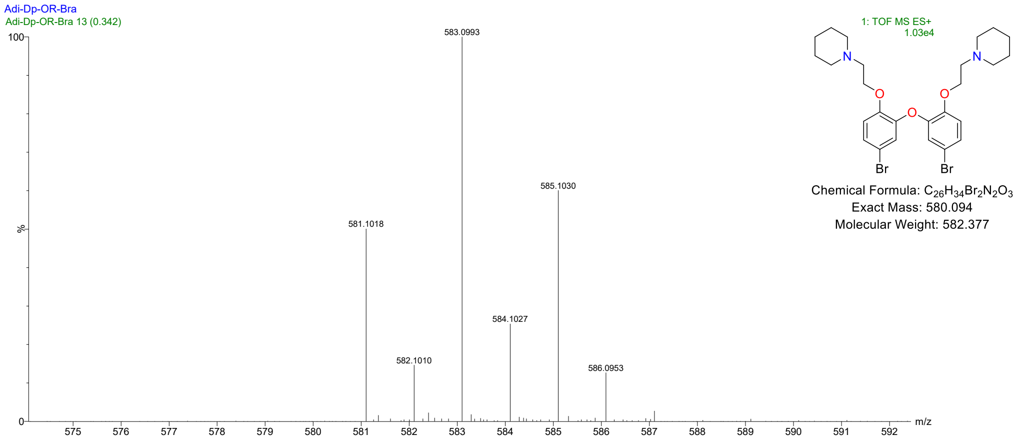

Figure S3. TOF-Mass spectrum (ES+) of compound **3**

Spectroscopic data for compound 5.

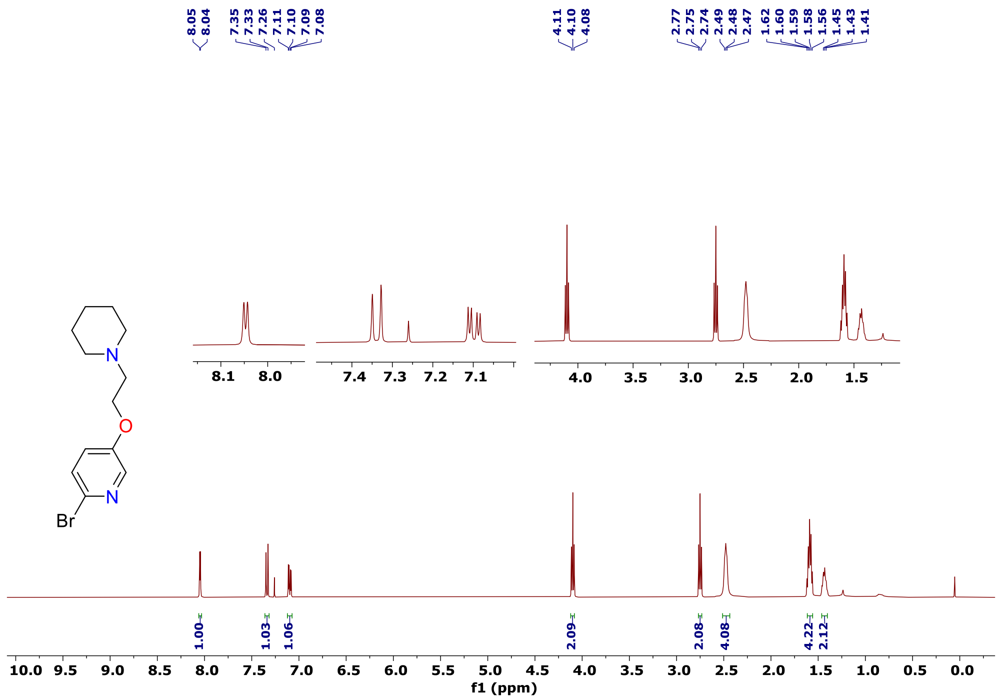

Figure S4. ^{1}H NMR spectrum in $CDCl_3$ of compound **5**

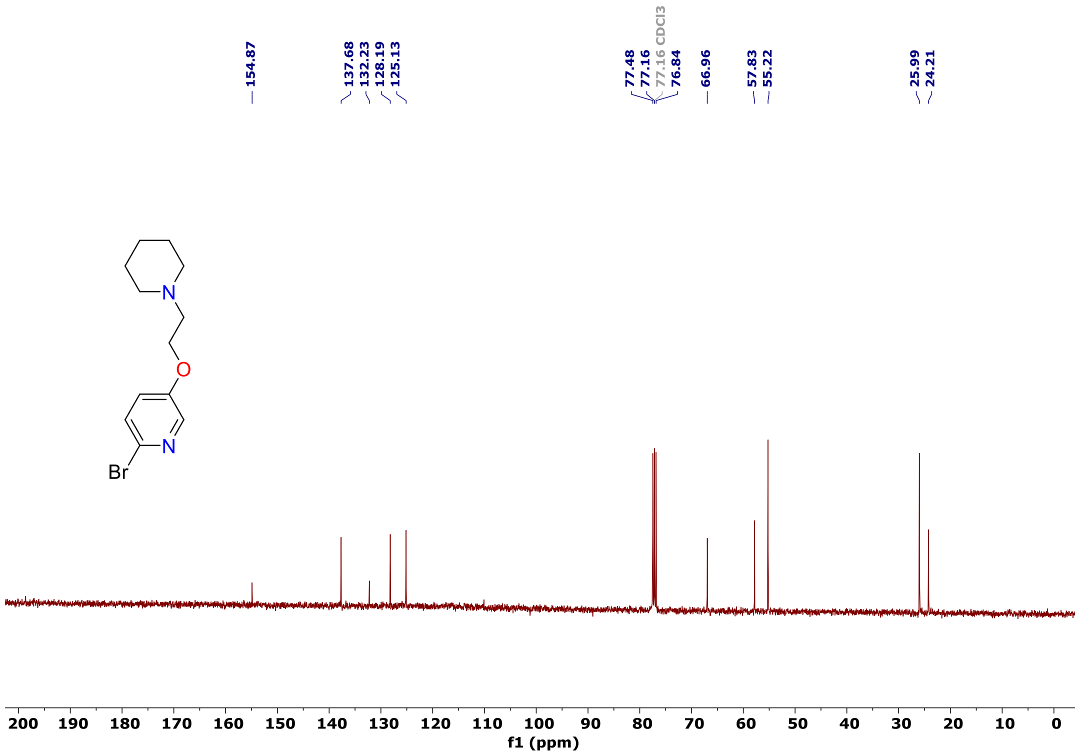

Figure S5. ^{13}C NMR spectrum in $CDCl_3$ of compound **5**

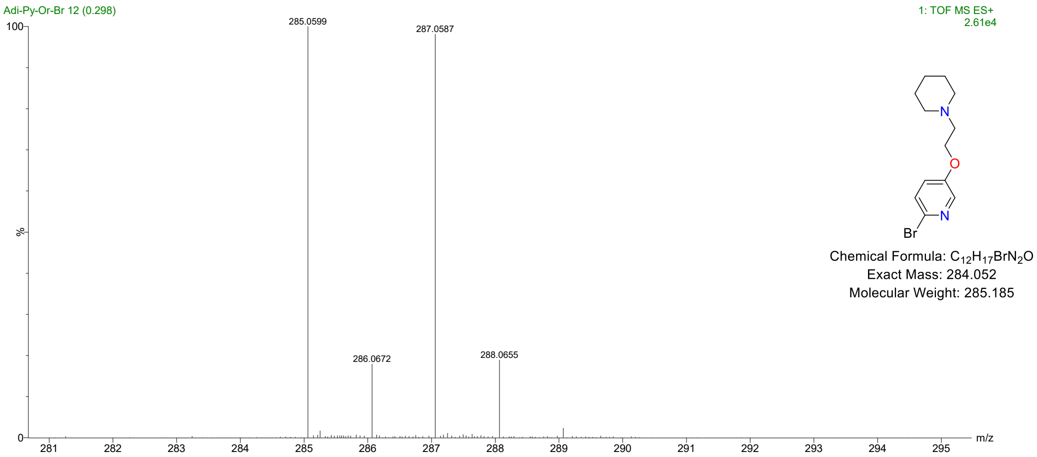

Figure S6. TOF-Mass spectrum (ES+) of compound **5**

Spectroscopic data for compound 6.

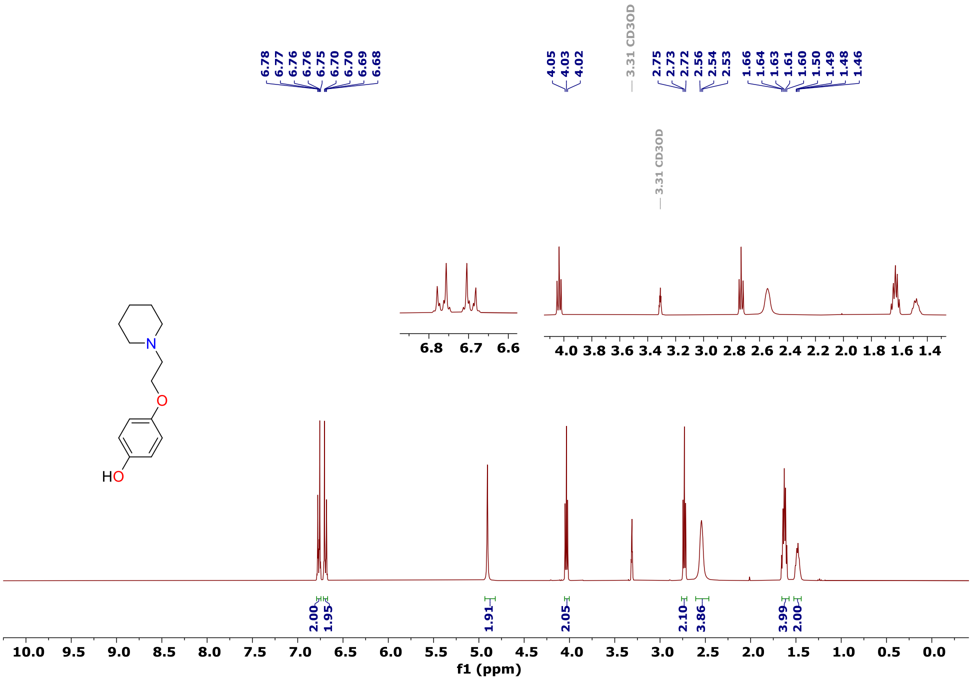

Figure S7. ^{1}H NMR spectrum in CD_3OD of compound **6**

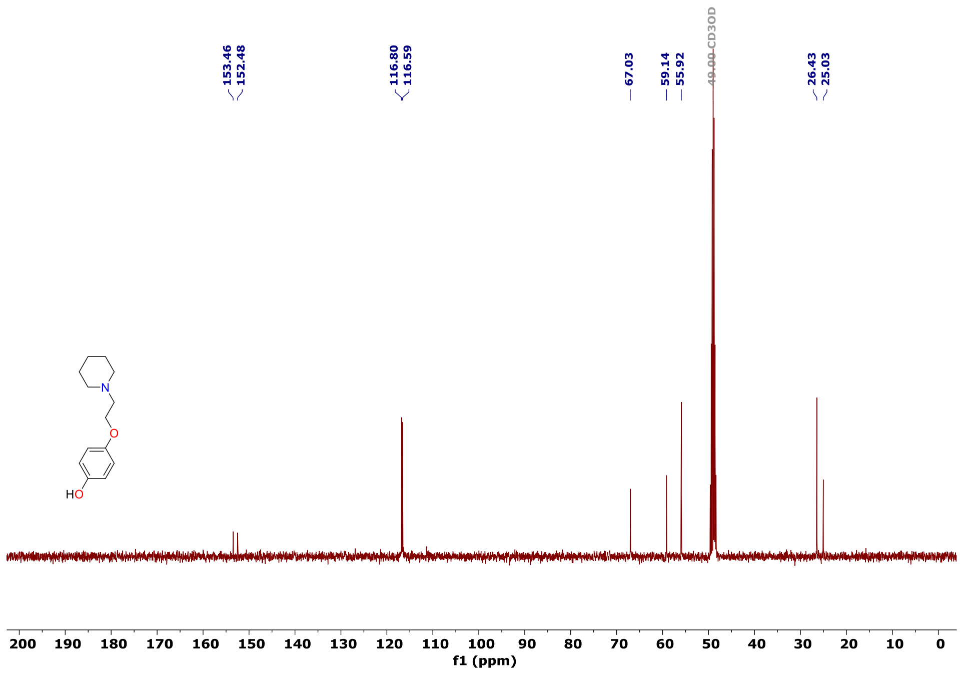

Figure S8. ^{13}C NMR spectrum in CD_3OD of compound **6**

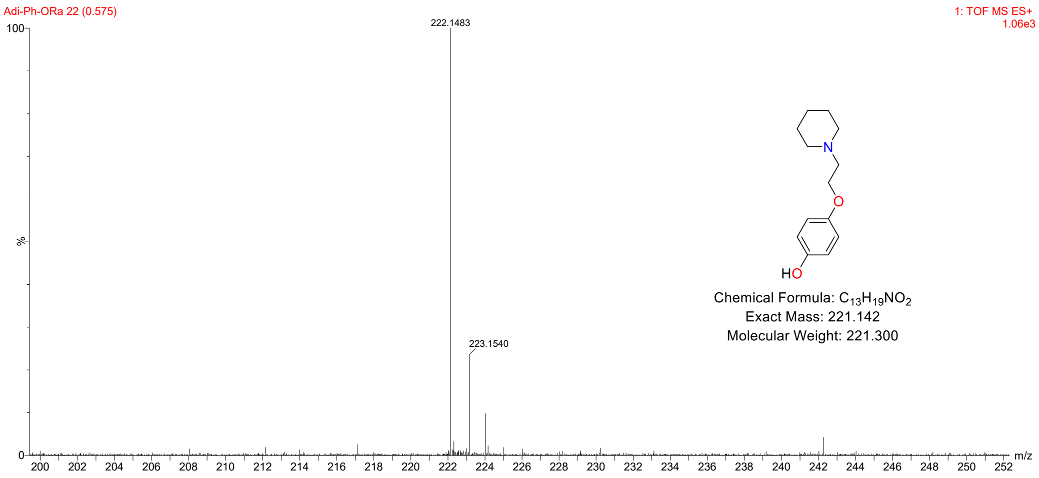

Figure S9. TOF-Mass spectrum (ES+) of compound **6**

Spectroscopic data for L[1].

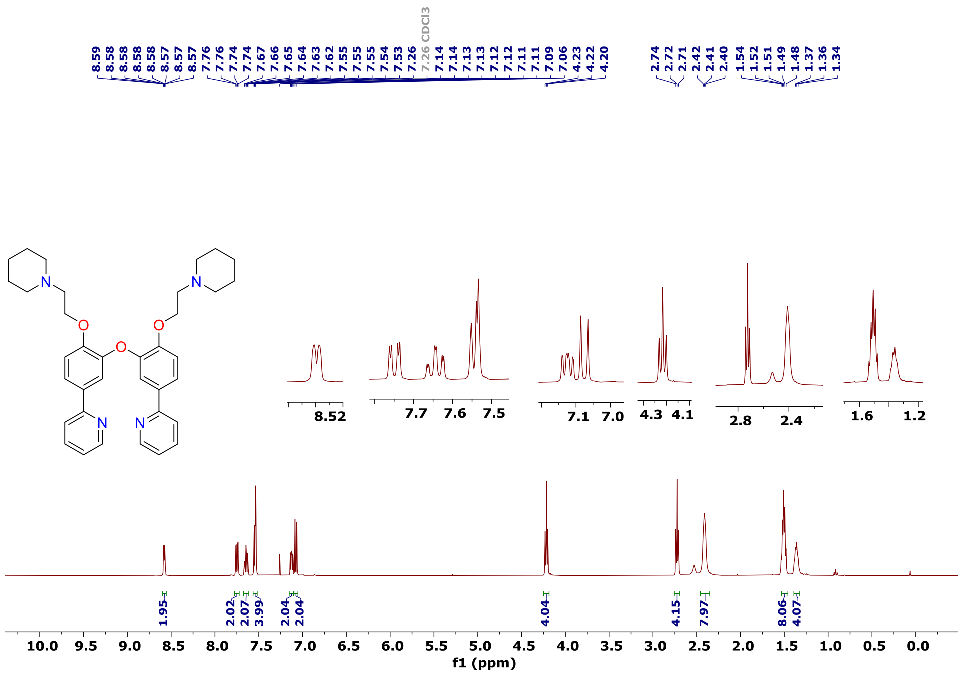

Figure S10. ^{1}H NMR spectrum in $CDCl_3$ of ligand **L[1]**

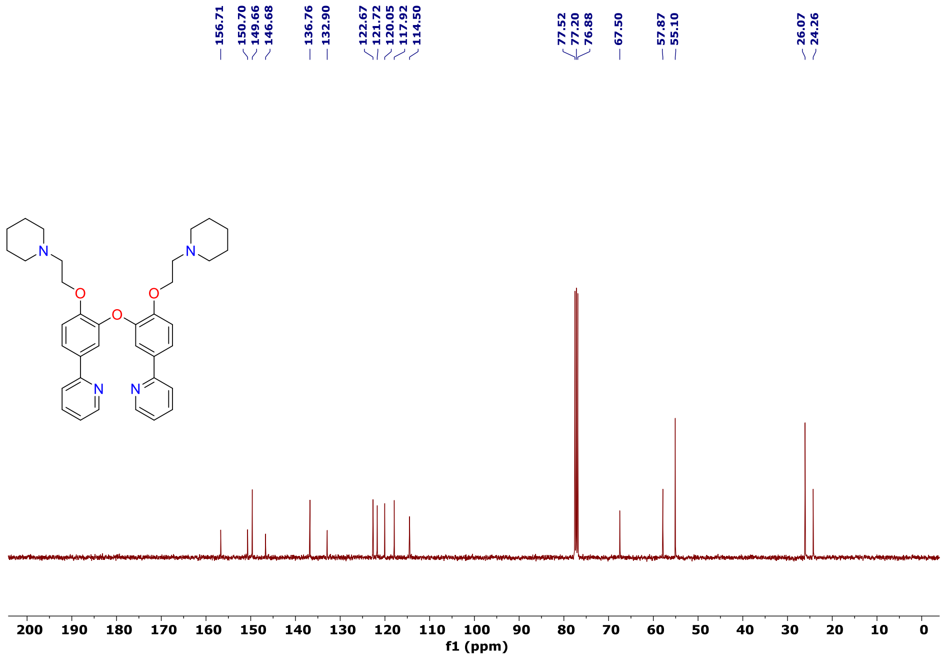

Figure S11. ^{13}C NMR spectrum in $CDCl_3$ of ligand **L[1]**

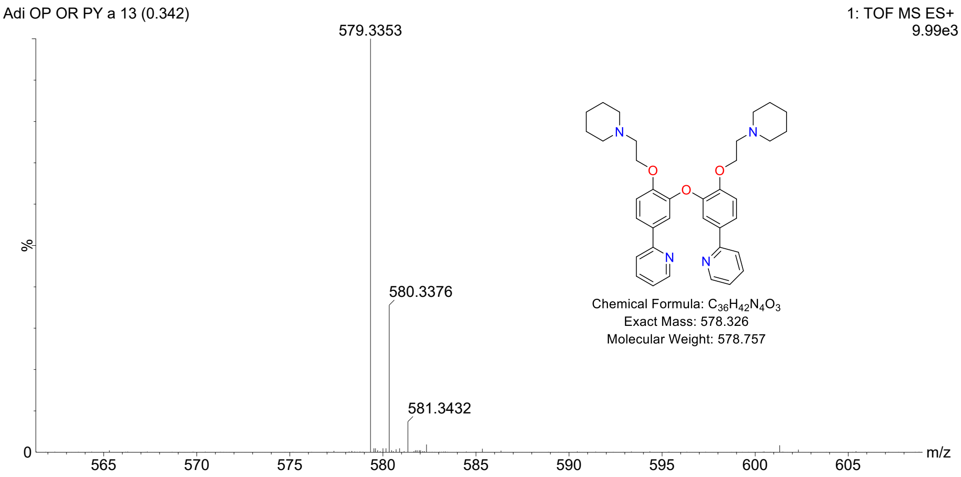

Figure S12. TOF-Mass spectrum (ES+) of ligand **L^1**

Spectroscopic data for L^2.

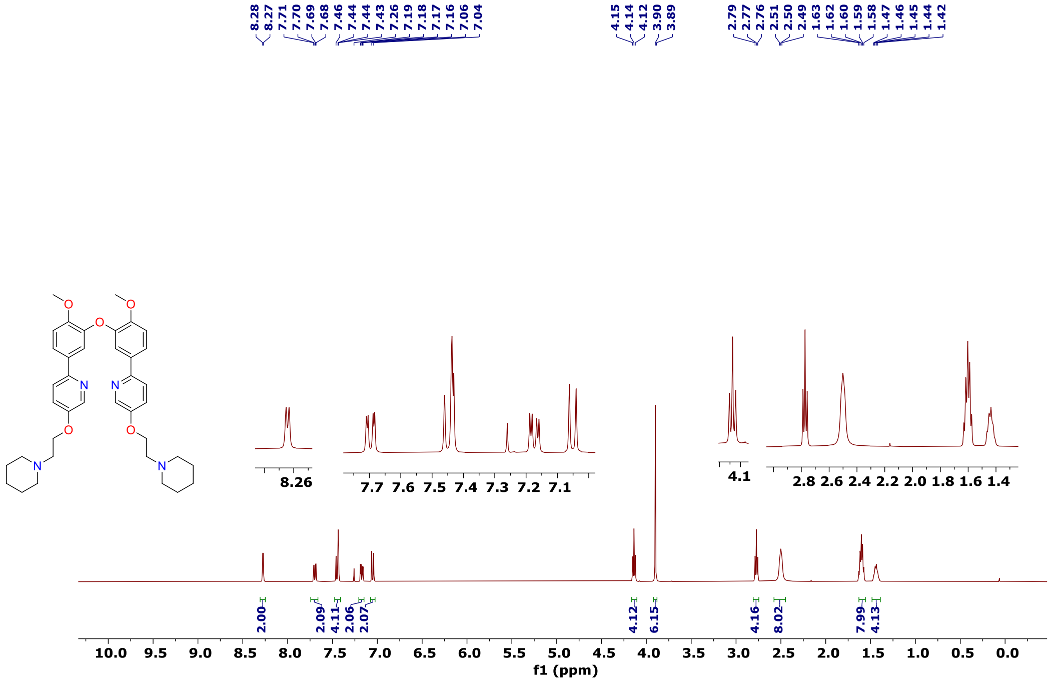

Figure S13. ^{1}H NMR spectrum in $CDCl_3$ of ligand **L^2**

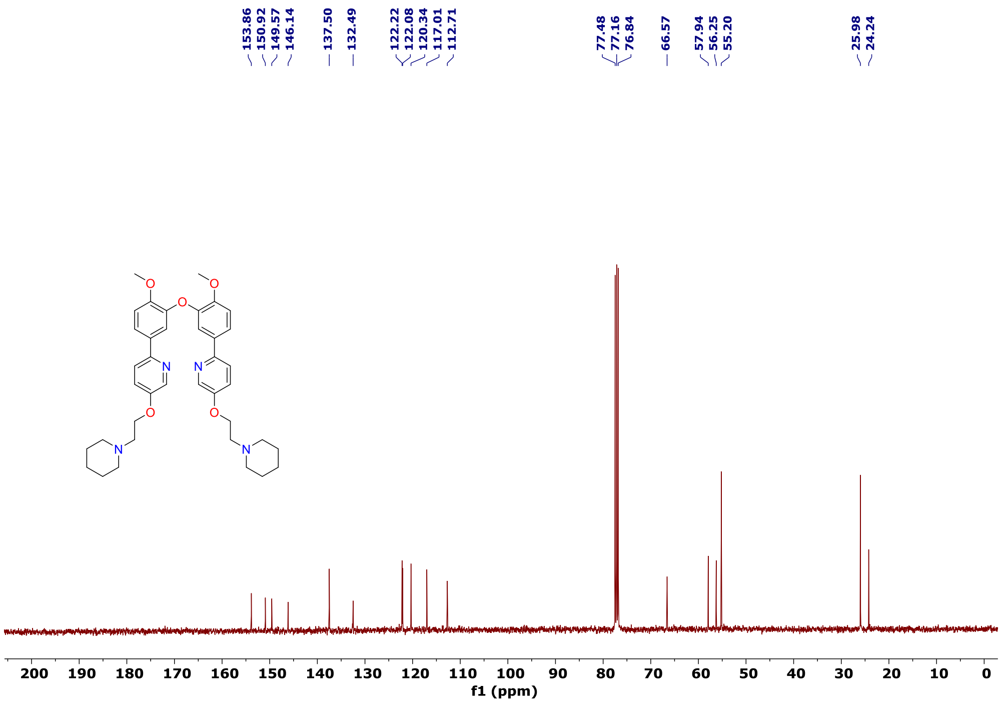

Figure S14. ^{13}C NMR spectrum in $CDCl_3$ of ligand **L**^{2}

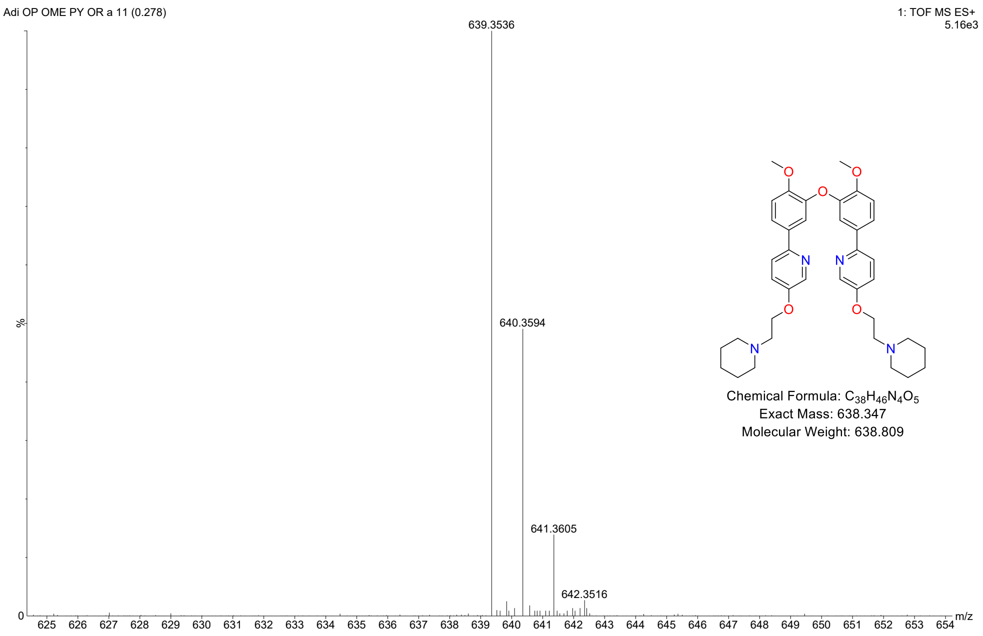

Figure S15. TOF-Mass spectrum (ES+) of ligand **L**^{2}

Spectroscopic data for L³.

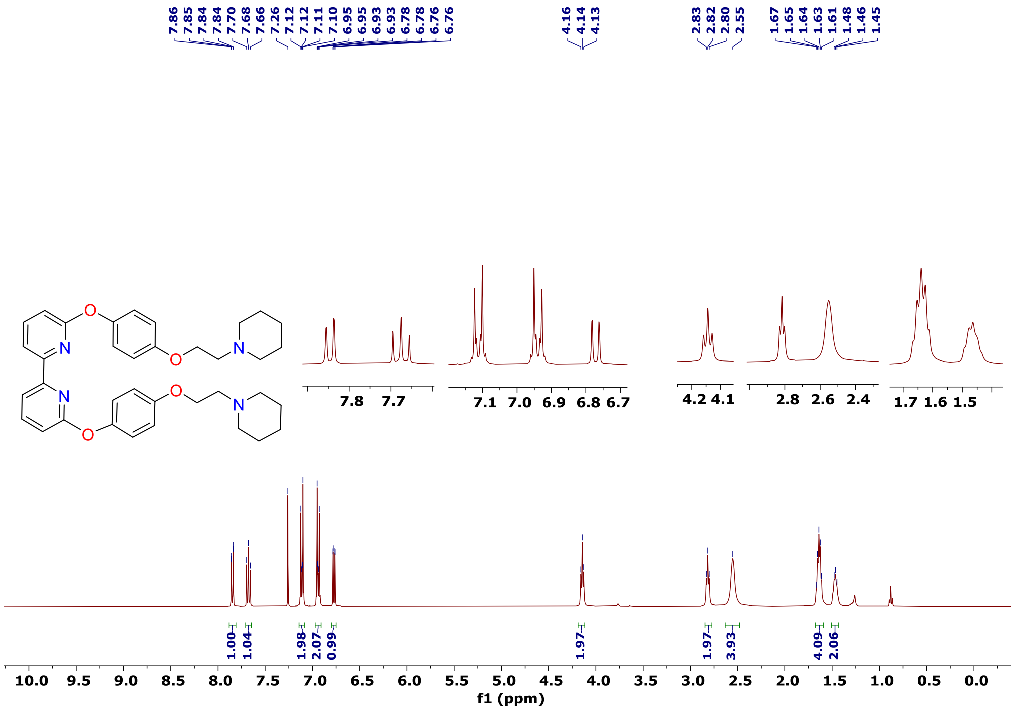

Figure S16. ^{1}H NMR spectrum in $CDCl_3$ of ligand **L³**

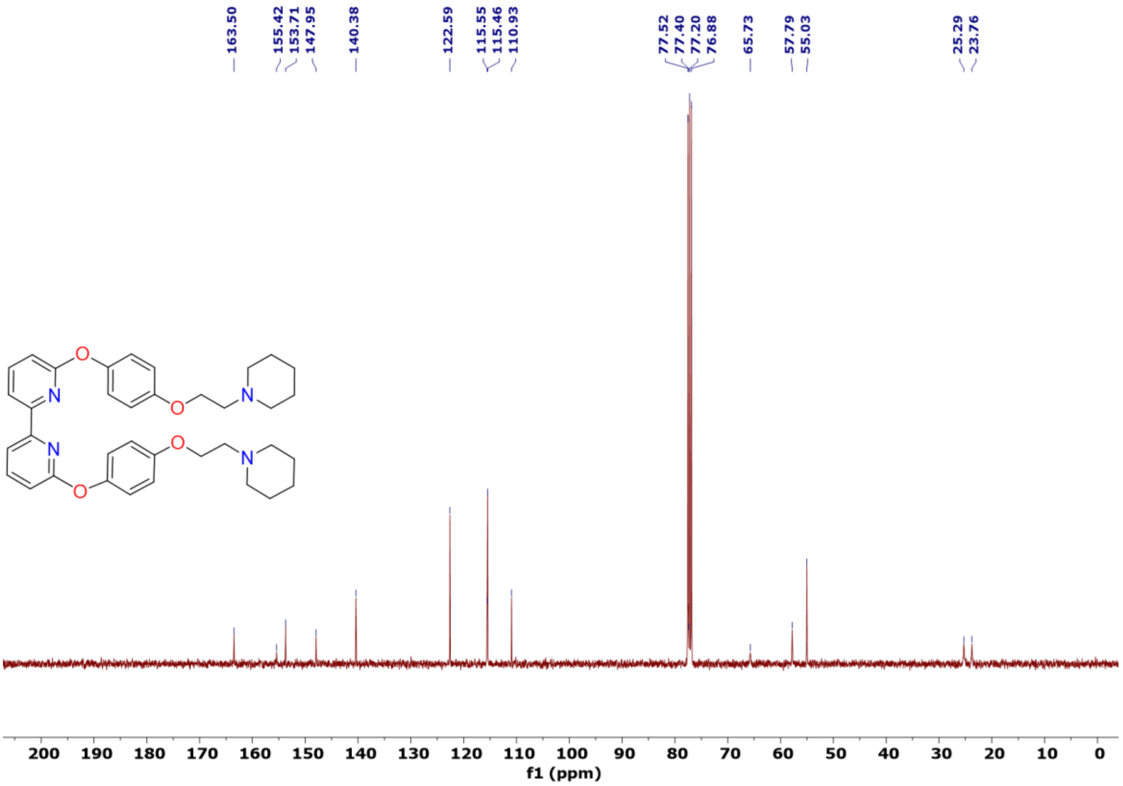

Figure S17. ^{13}C NMR spectrum in $CDCl_3$ of ligand **L³**

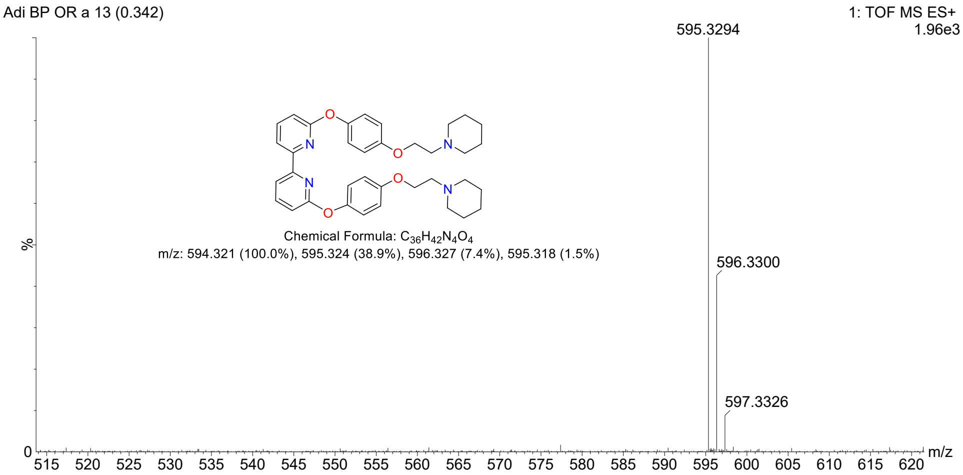

Figure S18. TOF-Mass spectrum (ES+) of ligand $\mathbf{L^3}$

Spectroscopic data for [Pt(L[1])].

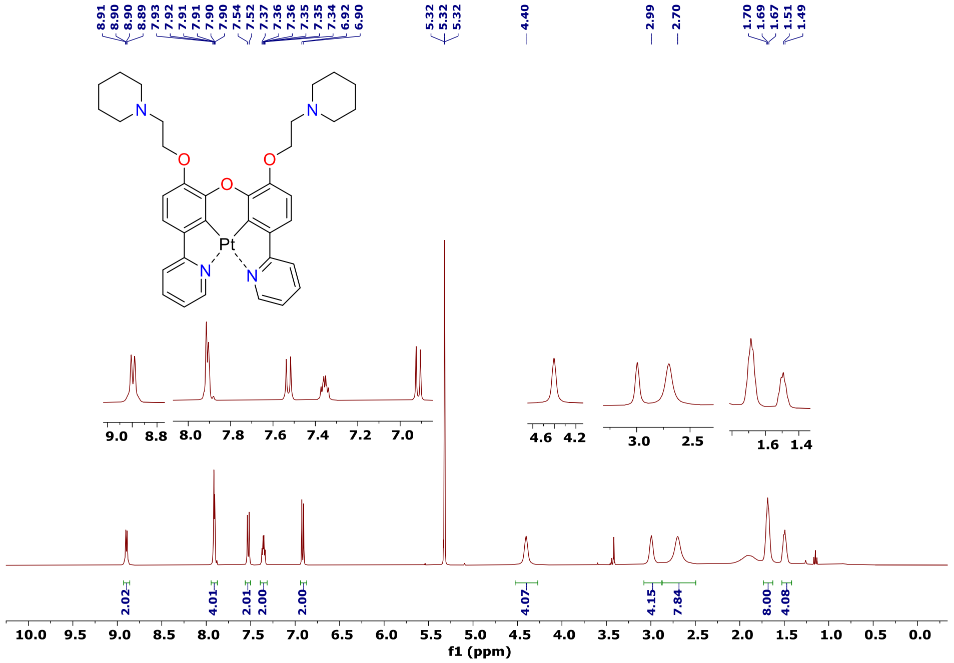

Figure S19. ^{1}H NMR spectrum in CD_2Cl_2 of [Pt($\mathbf{L^1}$)].

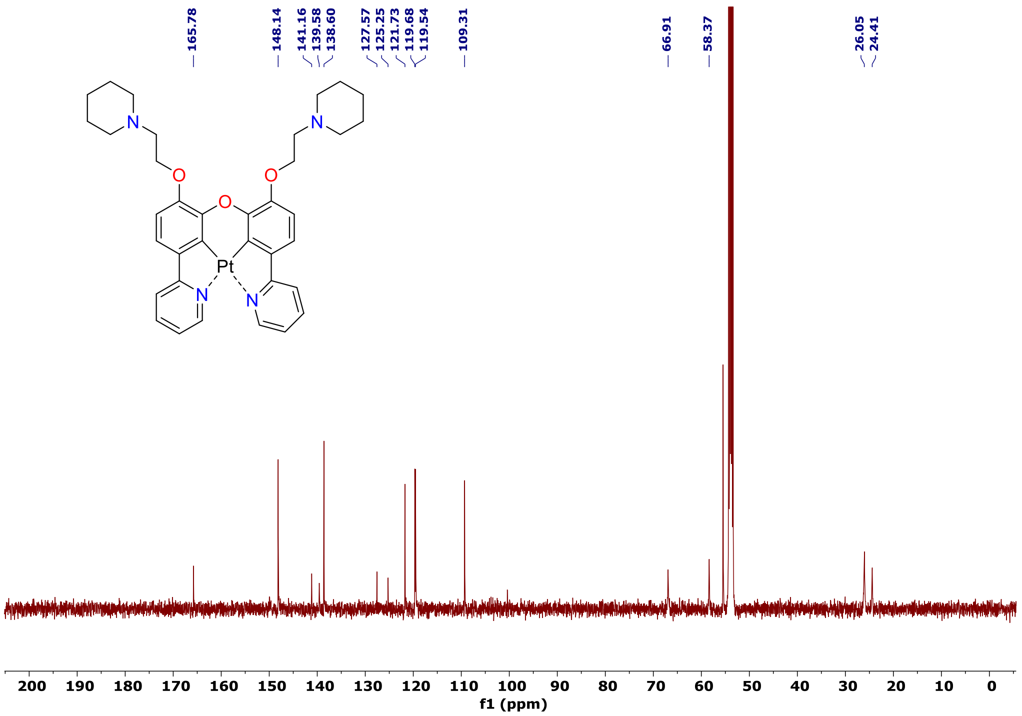

Figure S20. ^{13}C NMR spectrum in CD_2Cl_2 of [Pt($\mathbf{L^1}$)].

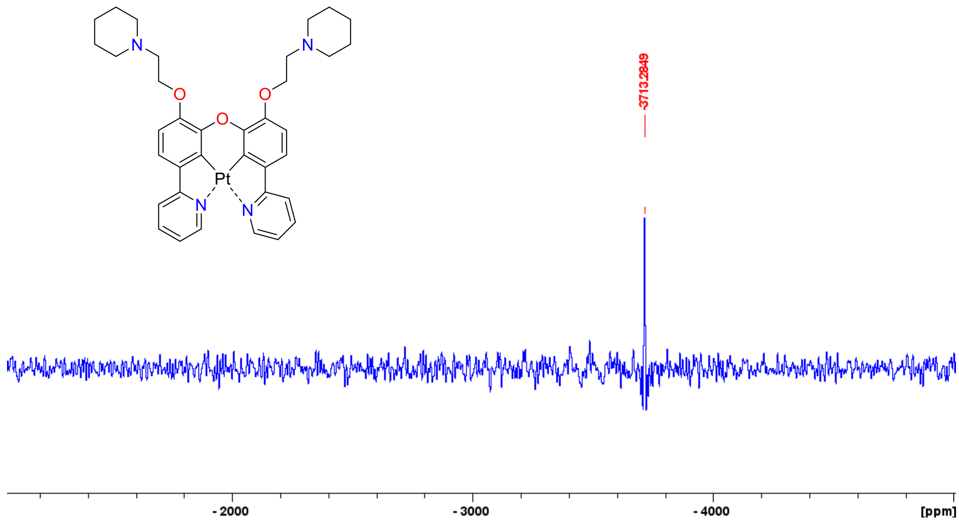

Figure S21. ^{195}Pt NMR spectrum in CD_2Cl_2 of [Pt($\mathbf{L^1}$)].

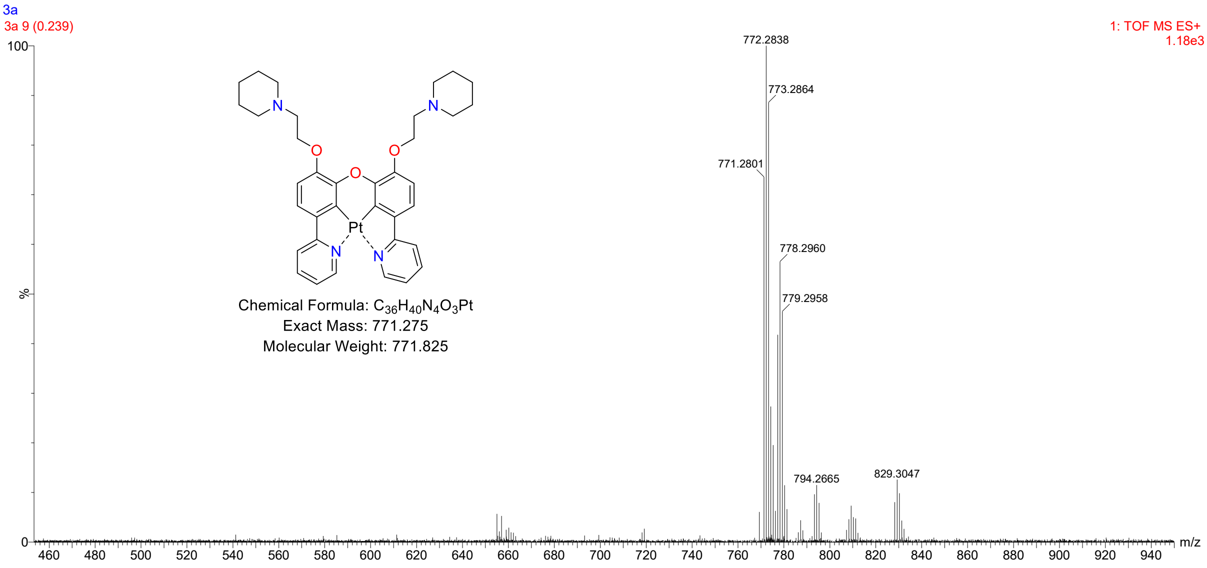

Figure S22. TOF-Mass spectrum (ES+) of [Pt(**L**[1])].

Spectroscopic data for [Pt(L[2])].

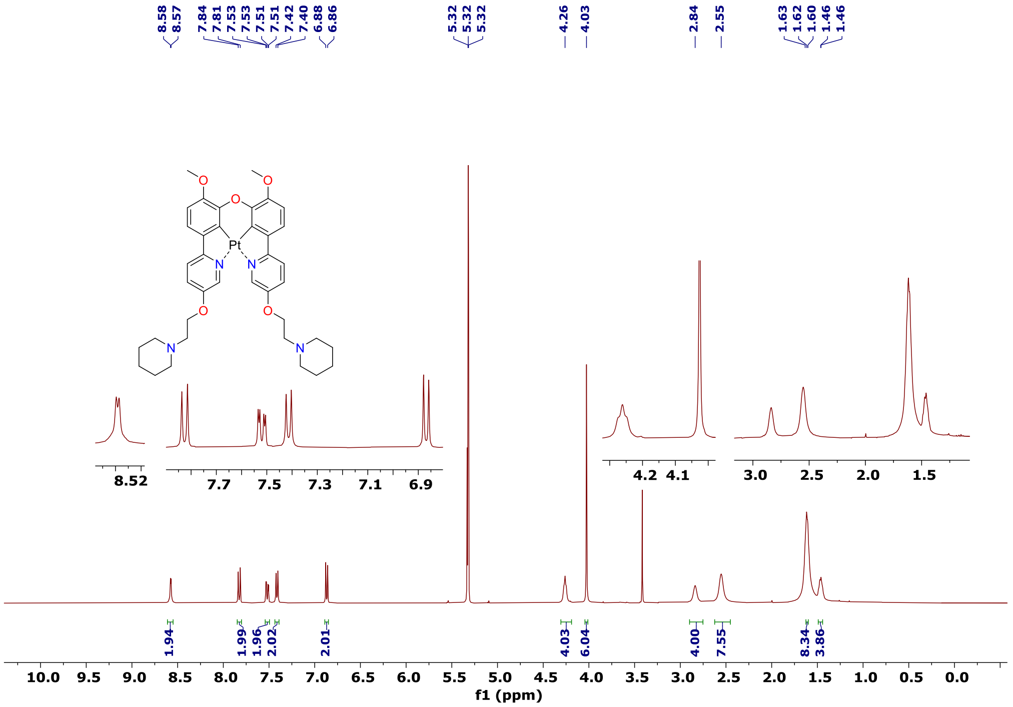

Figure S23. ^{1}H NMR spectrum in CD_2Cl_2 of [Pt(**L**[2])].

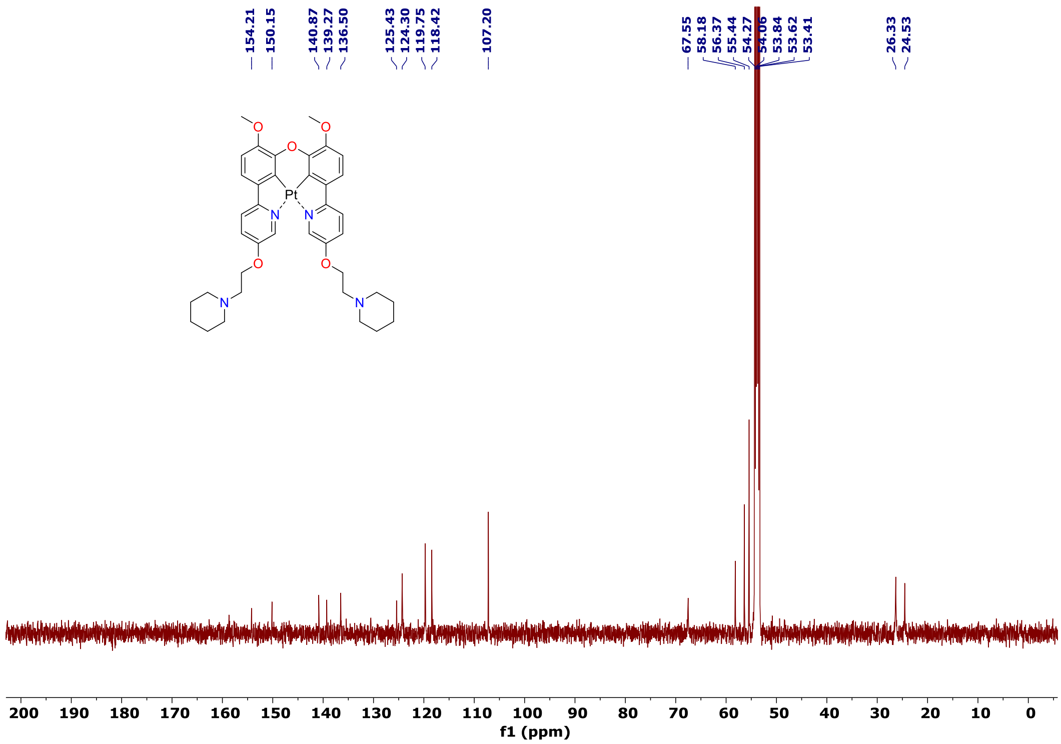

Figure S24. ^{13}C NMR spectrum in CD_2Cl_2 of [Pt(**L**^{2})].

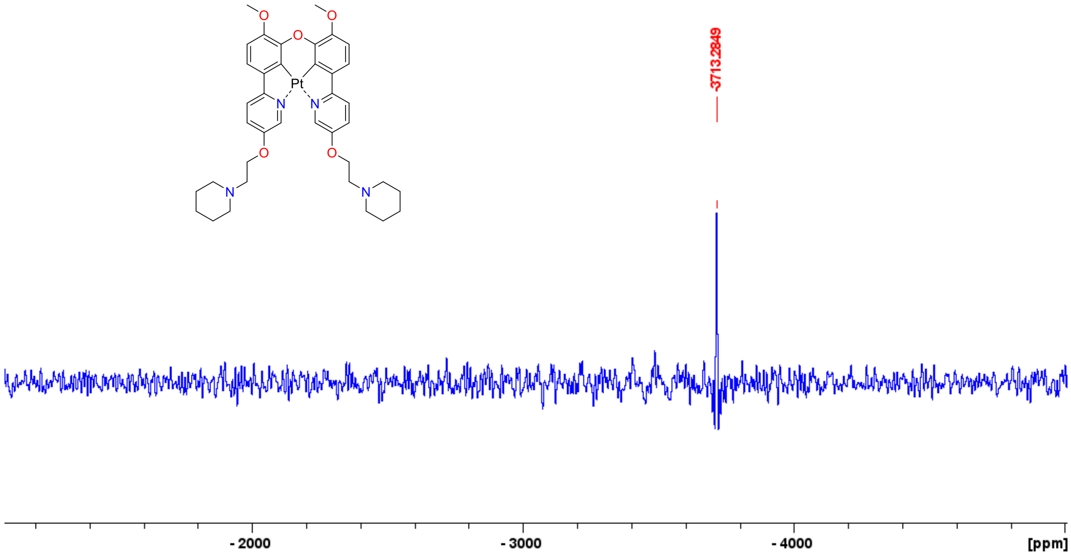

Figure S25. ^{195}Pt NMR spectrum in CD_2Cl_2 of [Pt(**L**^{2})].

Spectroscopic data for [Pt(L³)].

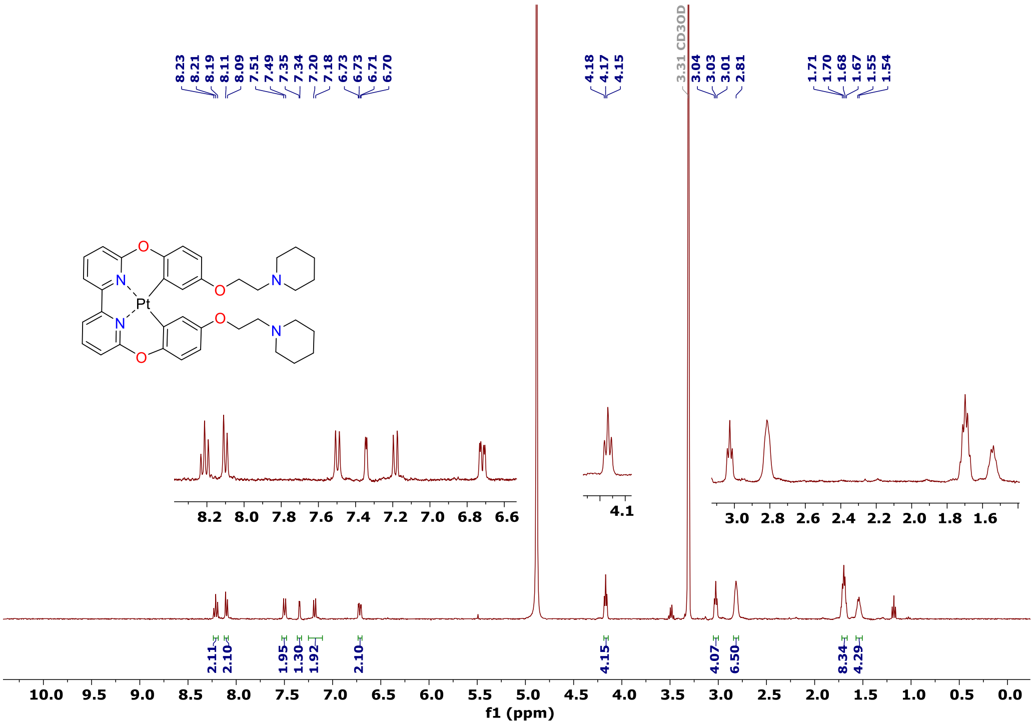

Figure S26. ^{1}H NMR spectrum in CD_3OD of [Pt(**L**^{3})].

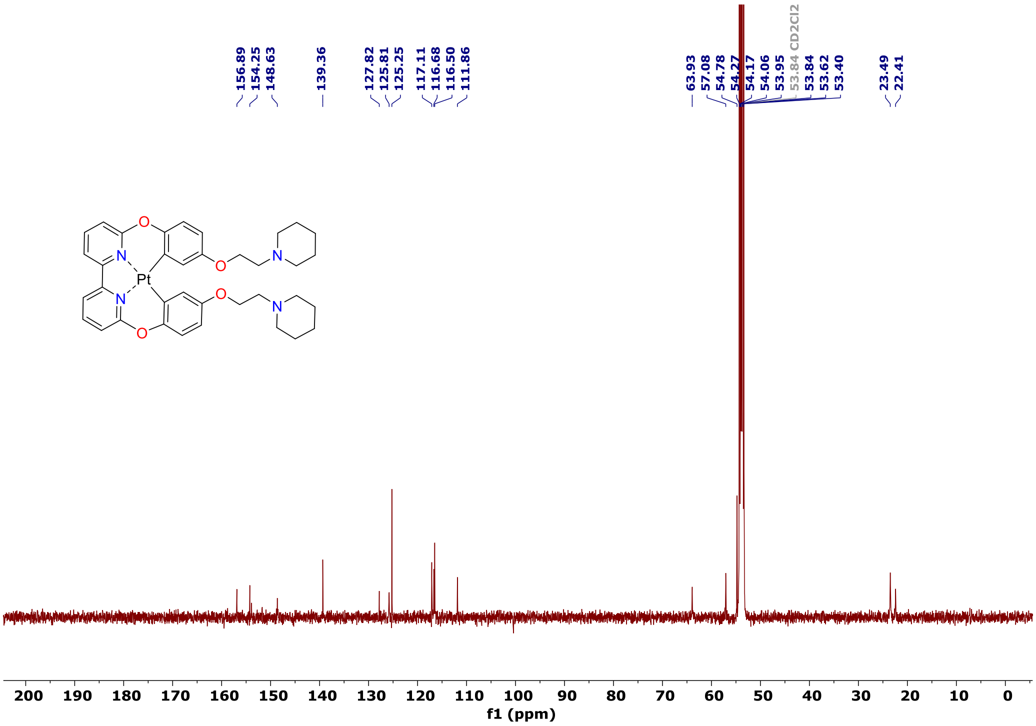

Figure S27. ^{13}C NMR spectrum in CD_2Cl_2 of [Pt(**L**^{3})].

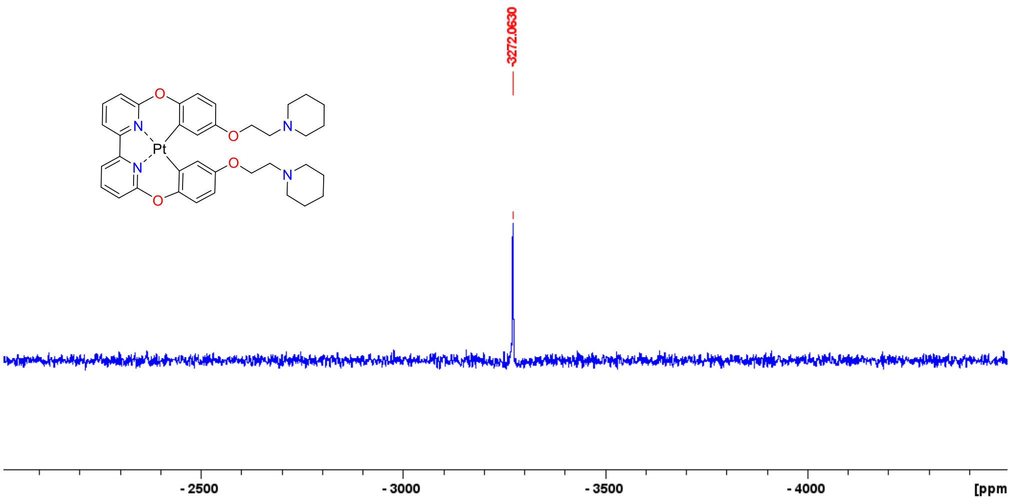

Figure S28. ^{195}Pt NMR spectrum in CD_3OD of [Pt(**L**^{3})].

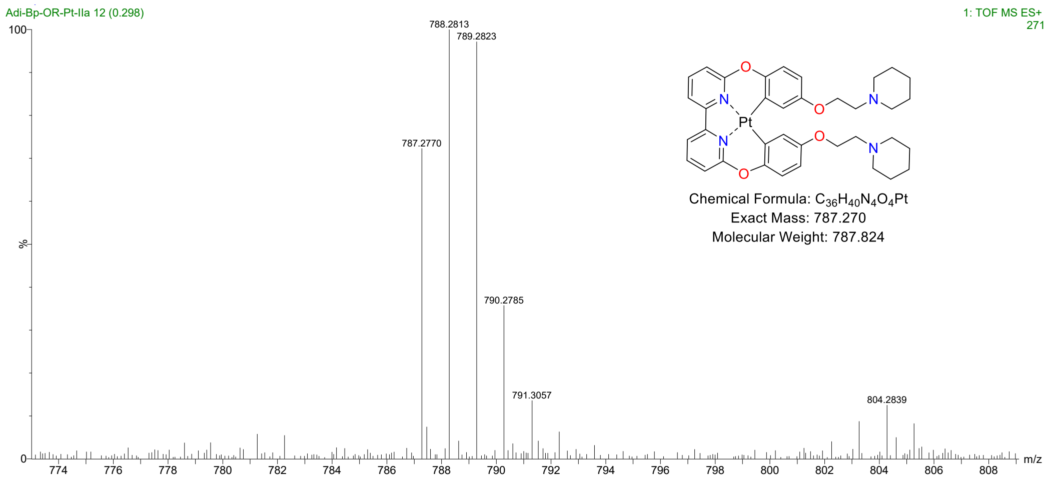

Figure S29. TOF-Mass spectrum (ES+) of [Pt(**L**^{3})].

DNA Preparation

G4 DNA sequences were purchased from Eurogentec or IDT and the duplex calf thymus DNA (ct-DNA) was purchased from Sigma-Aldrich. Stock solutions were prepared using Tris phosphate buffer (10 mM, pH 7.4) and the concentrations were determined by Agilent Cary UV 60 spectrometer. The stock solutions were then annealed at 95 °C for 5 minutes and cooled to room temperature overnight after the addition of KCl (100 mM).

**DNA**	**Sequence (5' to 3')**	**Molar extinction coefficient ($M^{-1}cm^{-1}$)**
**HTelo**	AGGGTTAGGGTTAGGGTTAGGG	228500
**cMyc**	TGAGGGTGGGTAGGGTGGGTAA	228700
**c-kit2**	CGGGCGGGCGCTAGGGAGGGT	205600
**ct-DNA**	NA	13200

Emission Titrations

Experiments were carried out using a BMG Clariostar Microplate reader with Greiner Bio-One half volume (100 μL/well) plates. Titrations were carried out using c-Myc G4 DNA, HTelo G4 DNA, c-kit2 G4 DNA, HRAS G4 DNA. CT-DNA and ST-DNA in a Tris phosphate buffer supplemented with KCl (10 mM, 100 mM KCl,pH 7.4). [Pt(**L**^{1})] and [Pt(**L**^{2})] concentrations were kept constant at 1 μM and the following G4 DNA equivalents were tested: 0, 0.1, 0.2, 0.4, 0.8, 1.6, 3.2 and 6.4 μM. For duplex, 10x base pair equivalents were used (i.e. 0, 1, 2, 4, 8, 16, 32 and 64 BPE).

Sample preparation was carried out by preparing stock solutions of double concentrations of the complex and highest concentration DNA, before carrying out a serial dilution with the DNAs and adding the complex. Plates were gently shaken for 5 minutes. Excitation was carried out at 380 nm and recorded from 450-600 nm. The changes in emission were plotted against the oligonucleotide concentration for each complex. Experiments were conducted in triplicate and binding constants were obtained by curve fitting PRISM using the equation below.

$$y = 0.5R[1/K + L + nx - [(1/K + L + nx)^{2} - 4Lnx]^{0.5}$$

Fluorescence response (y) was fitted against DNA concentration (x); R is the machine response, K is the association constant of ligand and DNA, L is the concentration of ligand and n is the number of binding sites per DNA. The number of binding sites per G4 unit was 2 for all cases of ligand-G4 DNA combinations apart from [Pt(**L**^{1})] with HRAS and c-myc, in which the number of binding sites was increased to 3 to obtain a fit with stable confidence intervals. The number of binding sits for the duplex DNA structures was calculated as 1 per ct-DNA BPE).

In vitro time-correlated single photon counting (TCSPC) studies

Time-resolved fluorescence decays were obtained using an DeltaFlex (Horiba) time-correlated single photon counting (TCSPC) instrument. A NanoLED 404 nm laser was chosen to excite the sample with the time windows in the range of 3-50 μs, depending on the sample's lifetime. A Ludox solution was set with a neutral density filter for recording the Instrument Response Function (IRF). The decays were monitored at λ_{em} = 507 nm. Tail fit and IRF omit were applied to the decay traces, before fitting by a bi-exponential mathematical iterative function:

$$I(t) = I_0 \left(a_1 \, e^{-\frac{t}{\tau 1}} + a_2 \, e^{-\frac{t}{\tau 2}} \right)$$

where the α1 and α2 are the contribution amplitude of the first (τ_1) and second(τ_2) components of lifetime. In the file of fitted results, the CHISQ value represents how well the model fits the decay curve; a smaller CHISQ means a higher convincing fit (normally < 1.5 is good).

The intensity-weighted average lifetime (τ_w) was calculated according to the fitted parameters using the function:

$$\tau_w = \frac{\tau_1^2 \, \alpha_1 + \tau_2^2 \, \alpha_2}{\tau_1 \, \alpha_1 + \tau_2 \, \alpha_2}$$

Geometry Optimization

The three platinum(II) complexes [Pt(**L**^{1})]- [Pt(**L**^{3})] were modelled. Initial 3D structures were generated from SMILES representations using RDKit, followed by geometry pre-optimization using the GFN2-xTB method. Final optimizations were performed at the DFT level using ORCA 6.1.0 with the B3LYP functional, def2-TZVP basis set, and Grimme's D4 dispersion correction. Solvent effects were modelled using the CPCM implicit solvation model with water as solvent.

Molecular Docking

Molecular docking studies were performed using AutoDock 4.2.6. DNA receptors included a G-quadruplex structure from the *c-myc* promoter region (PDB: 2L7V) and a B-form duplex DNA (PDB: 1XRW), with co-crystallized ligands removed prior to docking. Custom force field parameters for platinum (Rii = 2.75 Å, εii = 0.080 kcal/mol) were incorporated based on literature values for transition metals. Grid maps were generated to encompass the entire receptor surface. The Lamarckian Genetic Algorithm was employed with a population size of 150, 2,500,000 energy evaluations, mutation rate of 0.02, and crossover rate of 0.8. For each ligand–receptor combination, 30 independent docking runs were performed.

Absorption spectra.

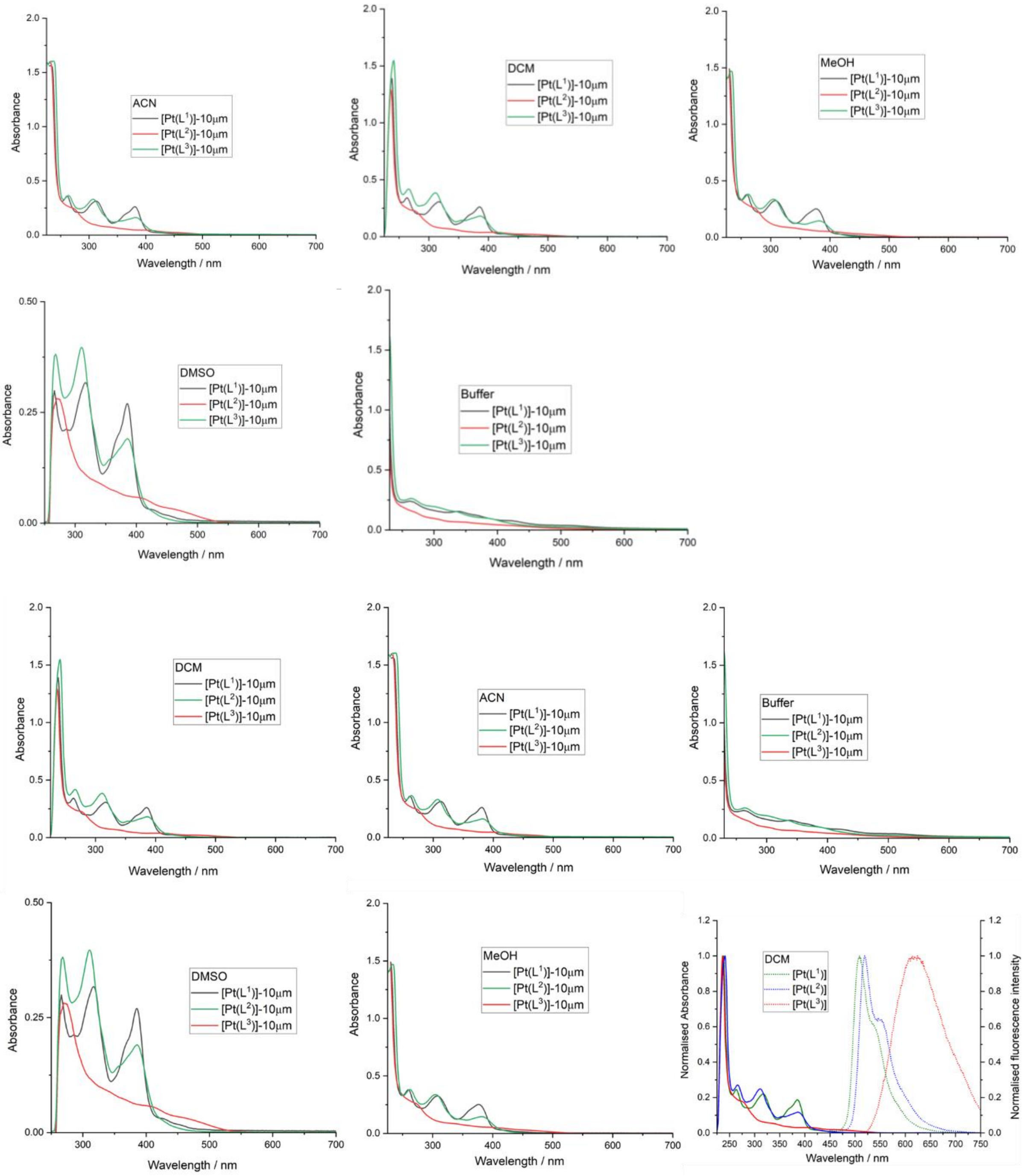

Figure S30. Absorbance spectra of [Pt(**L**^{1})], [Pt(**L**^{2})] and [Pt(**L**^{3})] in different solvents (ACN = CH_3CN and DCM = CH_2Cl_2).

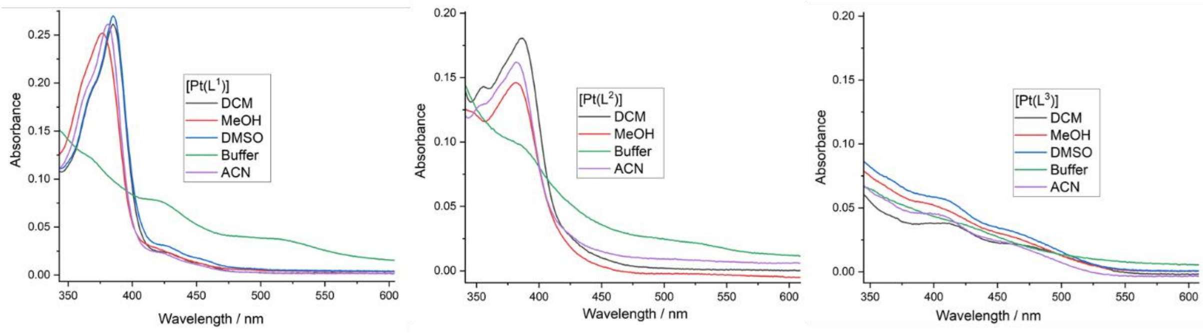

Figure S31. Expanded Absorbance spectra of [Pt(**L**[1])], [Pt(**L**[2])] and [Pt(**L**[3])] in different solvents.

Table S1 – UV/Vis and emission spectroscopic data for the platinum(II) complexes.

**Solvent**	**Pt(L**[1]**)-Abs λ_{max} (nm)**	**Pt(L**[2]**)-Abs λ_{max} (nm)**	**Pt(L**[3]**)-Abs λ_{max} (nm)**
**DCM**	238, 263, 317, 385, 427, 454	241, 267, 311, 355, 388	236, 275, 334, 410, 474
**MeCN**	233, 261, 313, 381, 423	235, 264, 308, 351, 384	232, 271, 337, 404, 464
**DMSO**	266, 316, 385, 427, 453, 492	268, 311, 385	270, 406, 461
**MeOH**	230, 260, 308, 377, 419	234, 262, 305, 347, 383, 522	230, 272, 335, 398, 463
**Buffer**	230, 265, 339, 367, 420, 514	230, 265, 299, 336, 384, 515	230, 267, 301, 345, 427

**Complexes**	**Emission λ_{max} (nm) in CH_2Cl_2**
[Pt(**L**[1])]	508, 539
[Pt(**L**[2])]	520, 552
[Pt(**L**[3])]	619

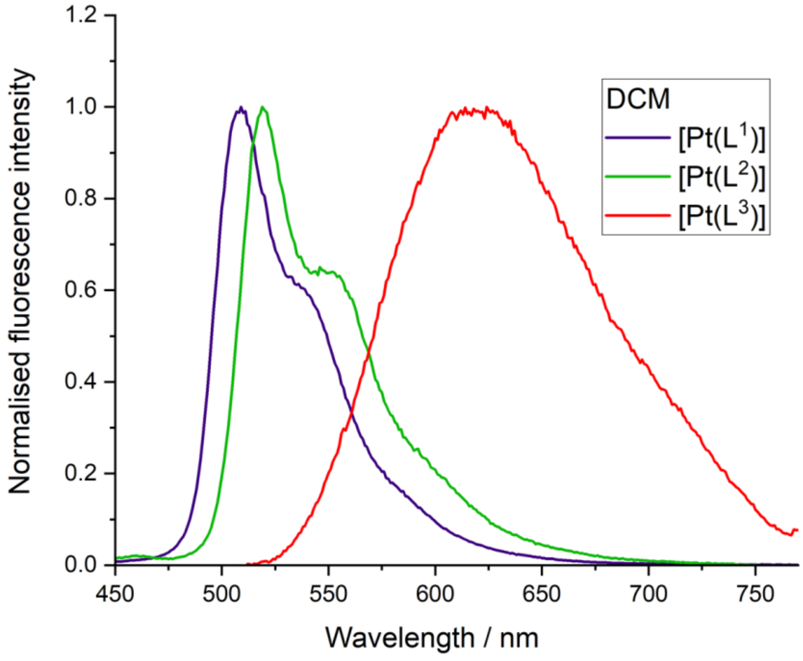

Figure S33. Normalised emission spectra (λex: 404 nm) of [Pt(**L**1)], [Pt(**L**2)] and [Pt(**L**3)], in CH_2Cl_2.

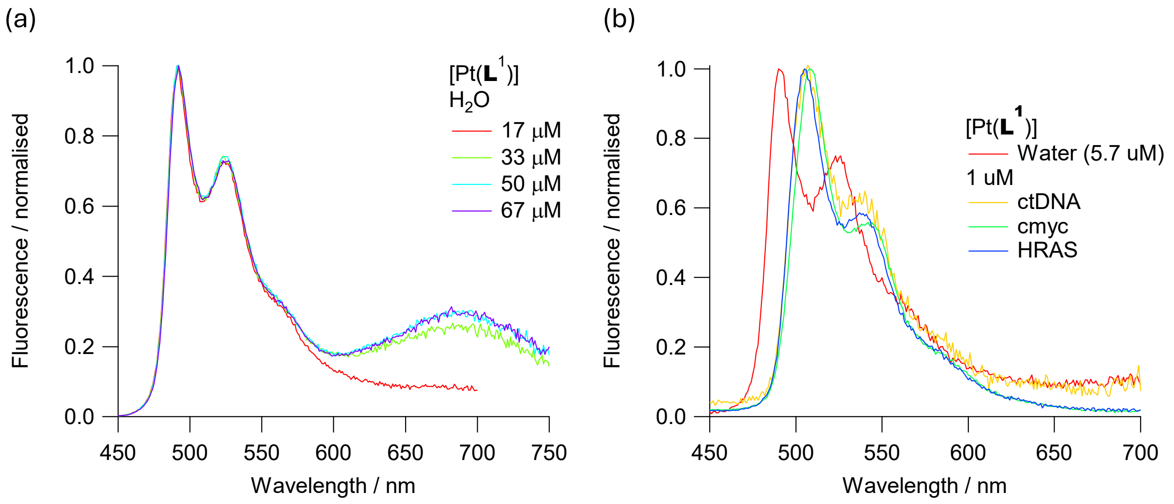

Figure S34. Normalised emission spectra (λex: 404 nm) of (a) different concentrations of [Pt(**L**1)] in aqueous buffer (solutions were prepared from DMSO stock solutions; % DMSO in the final solution being between 1.7 and 6.7%); a redshifted band (centred at ca. 680 nm) appears for probe concentrations above 30 µM indicating aggregation of the probe; (b) aqueous buffer solution of [Pt(**L**1)] (1 µM) in the absence and presence of G4 and duplex DNA; no redshifted band was observed indicating the absence of probe aggregation under this conditions.

Environmental sensitivity of [Pt(L¹)]

To test for possible factors affecting the lifetime of the complexes, apart from the DNA binding, we tested the lifetime of [Pt(**L**¹)] in solvents of different polarity and viscosity, as well as after degassing with nitrogen to remove residual oxygen from solutions.

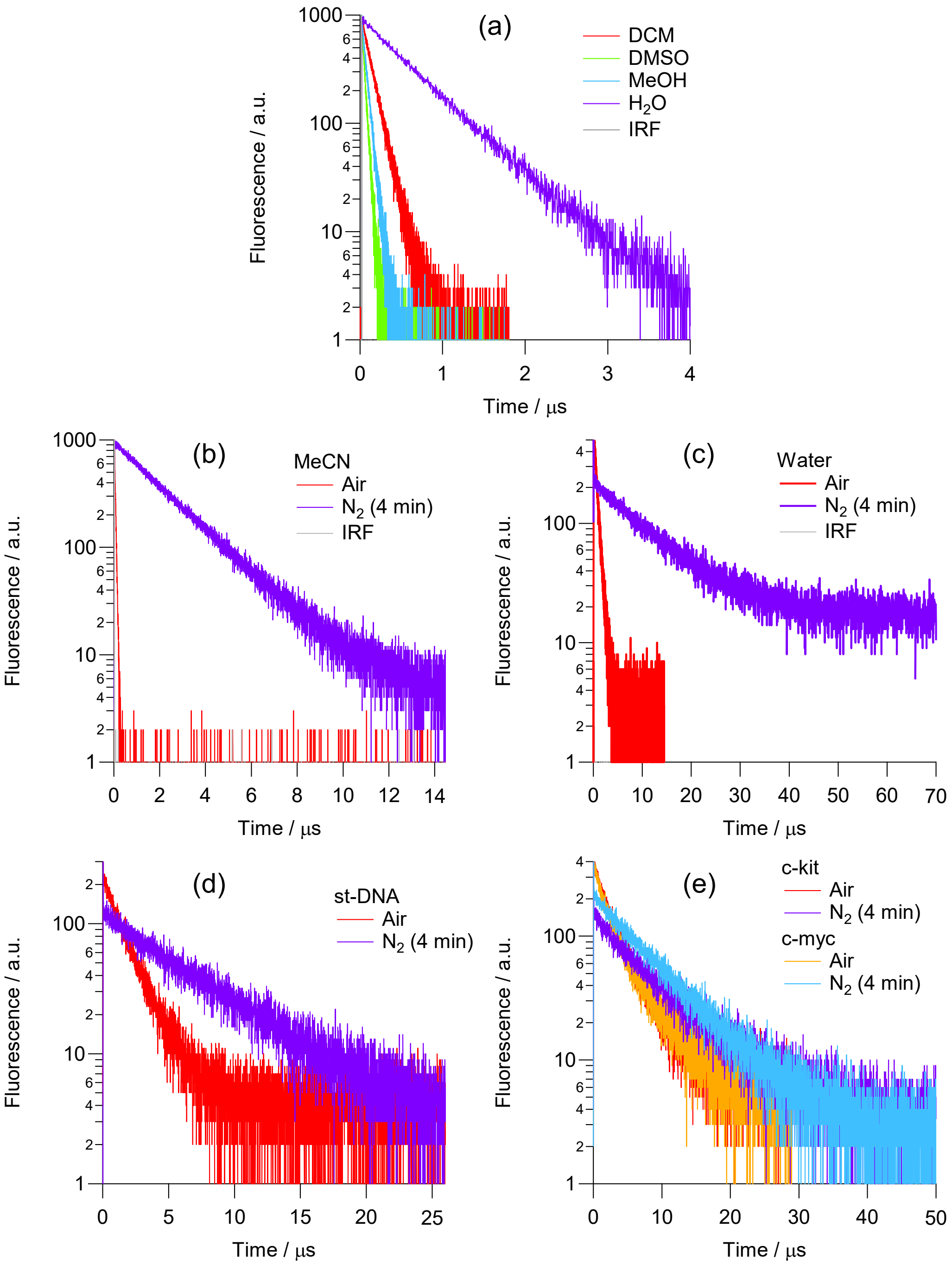

Figure S35. Fluorescence decays recorded for [Pt(**L**¹)] (a) in solvents of different polarity at standard aeration; and at standard aeration versus degassed conditions (b) in MeCN, (c) in water; (d) and in the presence of 50 µM st-DNA and (e) in the presence of c-kit and *c-myc* DNA under ambient and anaerobic conditions.

Varying polarity produced a small effect on the lifetime of the complex (Table S2), however, unexpectedly, water (the most polar solvent tested) was a clear exception from the trend, showing a very high lifetime. Otherwise, the data shows that polarity plays very minor role in the photophysics of this complex, and, therefore, electronic delocalisation within duplex/G4 is unlikely to be a dominant factor defining the lifetime.

Table S2. Fluorescence lifetimes (from monoexponential fits) recorded for [Pt(**L**^{1})] in solvents of different polarity

Solvent	Tau / μs
DCM	0.1202
DMSO	0.0346
MeCN	0.0380
MeOH	0.0548
H2O	0.5954

Next, we measured the phosphorescence lifetime of [Pt(**L**^{1})] in aqueous buffered solutions in the absence and presence of DNA (duplex and G4) at ambient oxygen pressure, and after 10 min of bubbling with nitrogen, leading to oxygen removal. It could be seen that the lifetime in water is the shortest, followed by that of a duplex-–bound form, then the G4-bound form (Table S3). However, in all cases upon bubbling N_2 the lifetime saturates at ca 7 μs, giving evidence that protection from oxygen by the DNA loops and groves is the dominant mechanism of the light switch effect upon binding to DNA.

Table S3. Fluorescence lifetimes (from monoexponential fits) recorded for [Pt(**L**^{1})] in water and in the presence of 50 μM st-DNA and G4s (*c-kit* and *c-myc*) DNA under ambient and anaerobic conditions (10 min bubbling with N_2).

	Tau / μs	
	Air	N_2
MeCN	0.038	2.144
H_2O	0.611	6.767
st-DNA	1.590	6.114
c-kit	3.256	7.430
c-myc	3.256	7.312

Finally, to assess the potential effect of viscosity, we recorded the emission of [Pt(**L**[1])] (i.e. the complex used to perform the detailed cellular imaging) in water:glycerol mixtures. Increasing % glycerol (i.e. higher viscosity) led to a very significant increase in lifetime of the probe. However, we hypothesised that the dominant feature of solutions with high % glycerol is poor dioxygen solubility. Therefore, we bubbled glycerol solution of [Pt(**L**[1])] with nitrogen and measured a significant increase in lifetime as a result (to ca 7μs), while bubbling with oxygen reduced the lifetime (Table S4). This data confirms that solvent viscosity alone, leading to the rigidification of the structure, is not sufficient to alleviate oxygen quenching leading to shorter lifetimes.

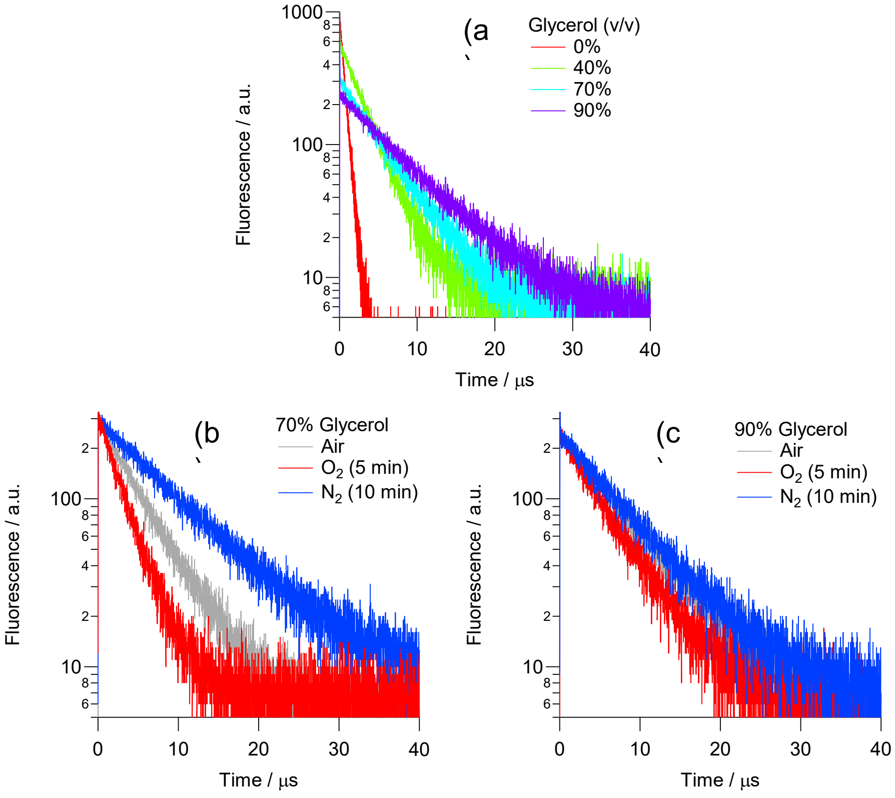

Figure S36. Fluorescence decays recorded for [Pt(**L**[1])] (a) in binary mixtures of glycerol/water of different content under ambient conditions and under O_2 and N_2-saturated conditions in binary mixtures with (b) 70% and (c) 90% glycerol (by volume) .

Table S4. Fluorescence lifetimes (from monoexponential fits) recorded for [Pt(**L**^{1})] in solvents of different viscosity, obtained by mixing glycerol and water, under ambient and anaerobic conditions.

	Tau / μs	
Glycerol (%)	Air-saturated	N_2-saturated
0	0.611	6.767
40	2.884	-
70	4.956	8.887
90	7.163	7.561

DNA binding studies

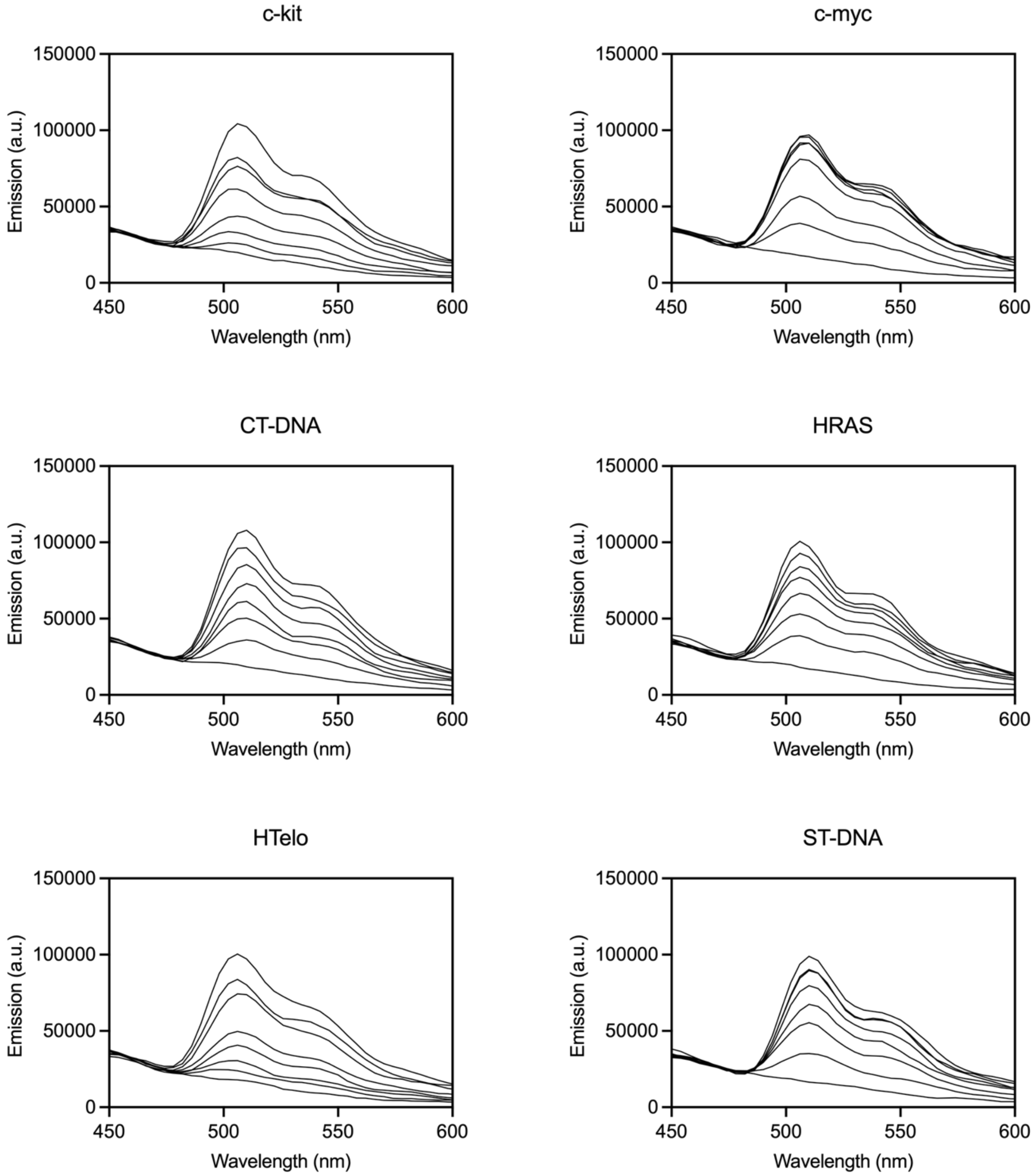

Figure S37. DNA emission titrations with [Pt($\mathbf{L}^1$)] against a range of DNA topologies. Concentration of compound was kept constant at 1 μM and DNA concentrations tested at 0, 0.1, 0.2, 0.4, 0.8, 1.6, 3.2 and 6.4 μM for G4 topologies, and 10x BPE for duplex topologies. Excitation was carried out at 380 nm, and emission was collected between 450-600 nm.

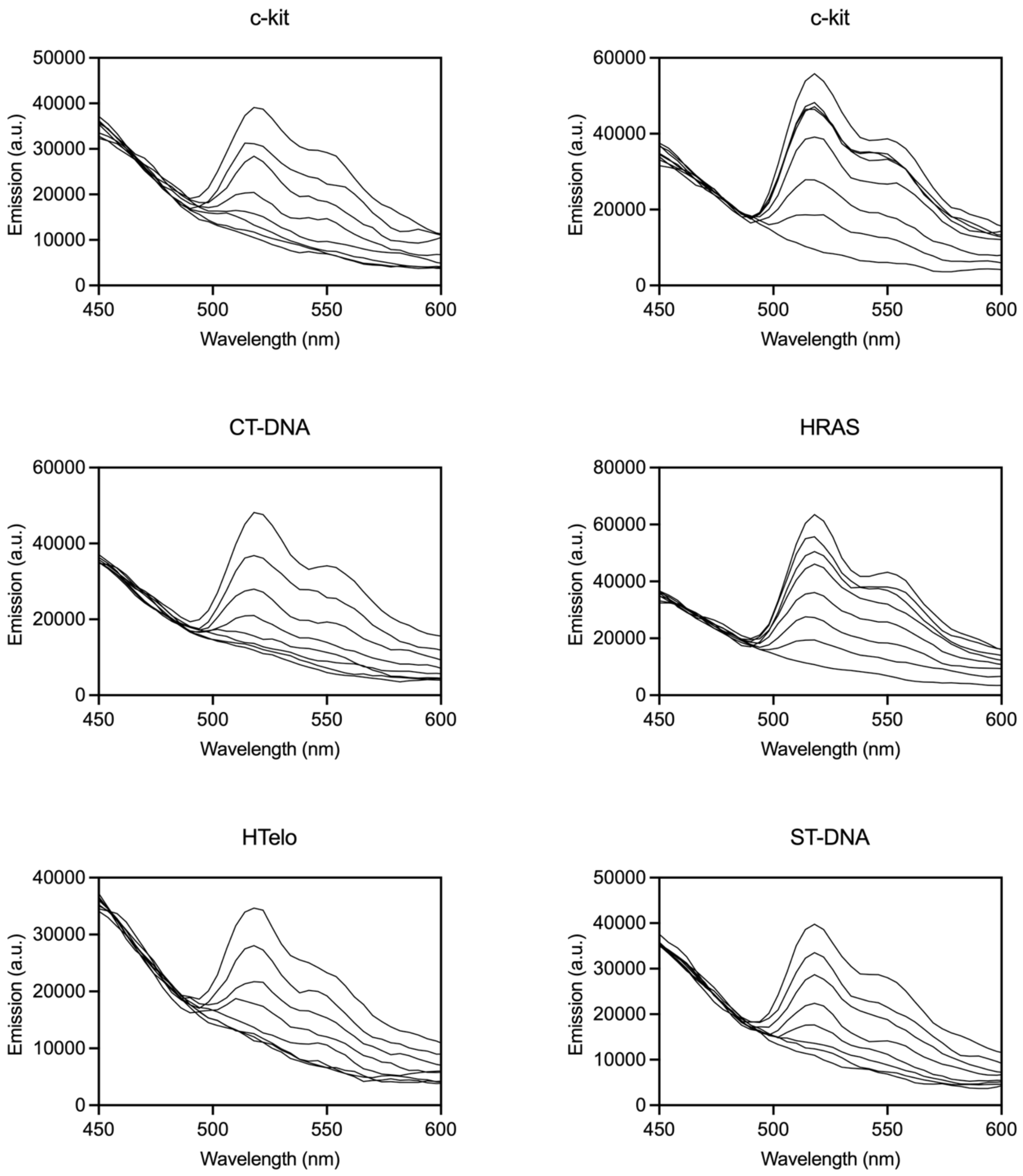

Figure S38. DNA emission titrations with [Pt(L^2)] against a range of DNA topologies. Concentration of compound was kept constant at 1 µM and DNA concentrations tested at 0, 0.1, 0.2, 0.4, 0.8, 1.6, 3.2 and 6.4 µM for G4 topologies, and 10x BPE for duplex topologies. Excitation was carried out at 380 nm, and emission was collected between 450-600 nm.

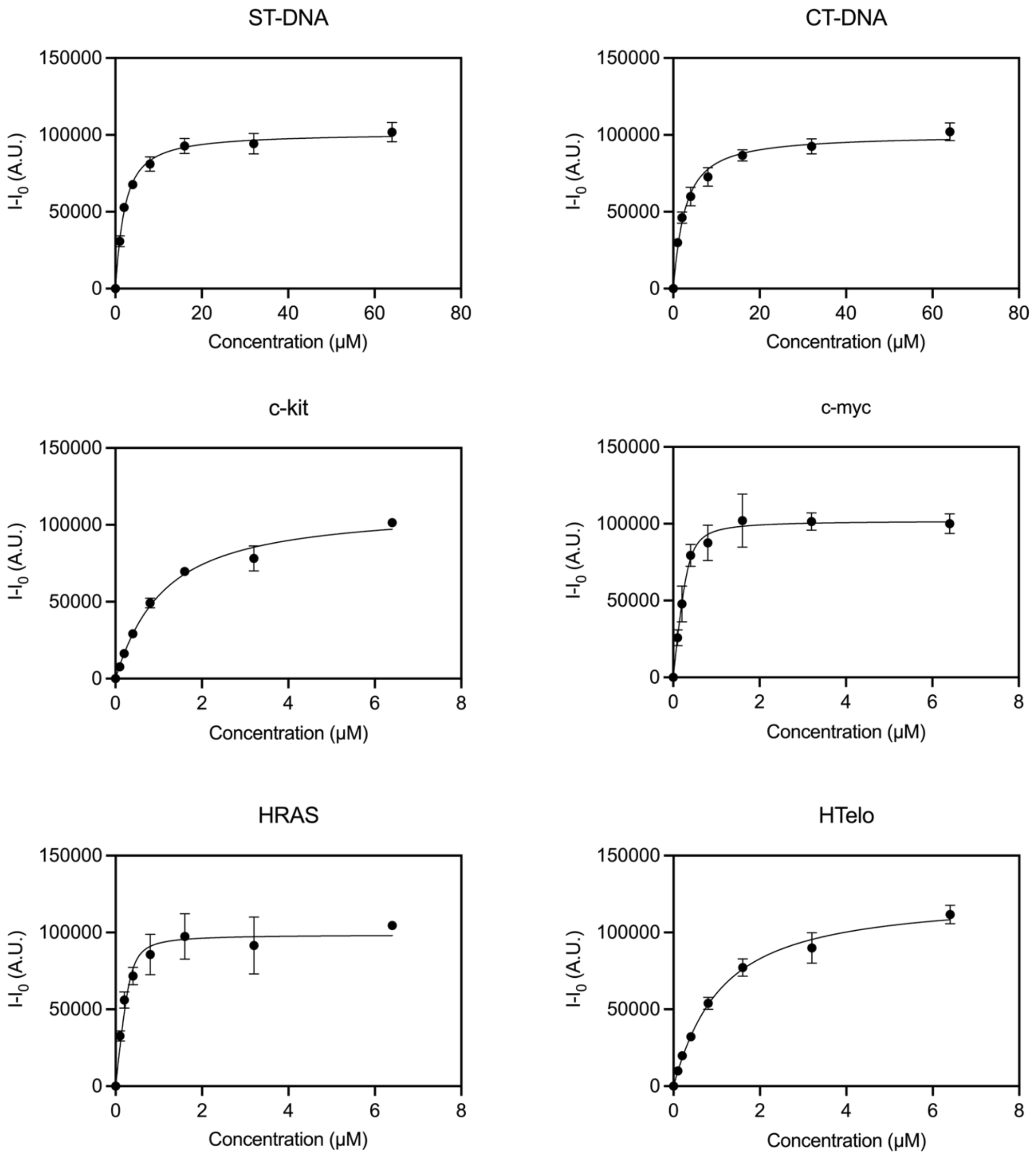

Figure S39. DNA emission titrations with [Pt(**L**^{1})] against a range of DNA topologies. Concentration of compound was kept constant at 1 μM and DNA concentrations tested at 0, 0.1, 0.2, 0.4, 0.8, 1.6, 3.2 and 6.4 μM for G4 topologies, and 10x BPE for duplex topologies. Excitation was carried out at 380 nm and emission collected at 510 nm.

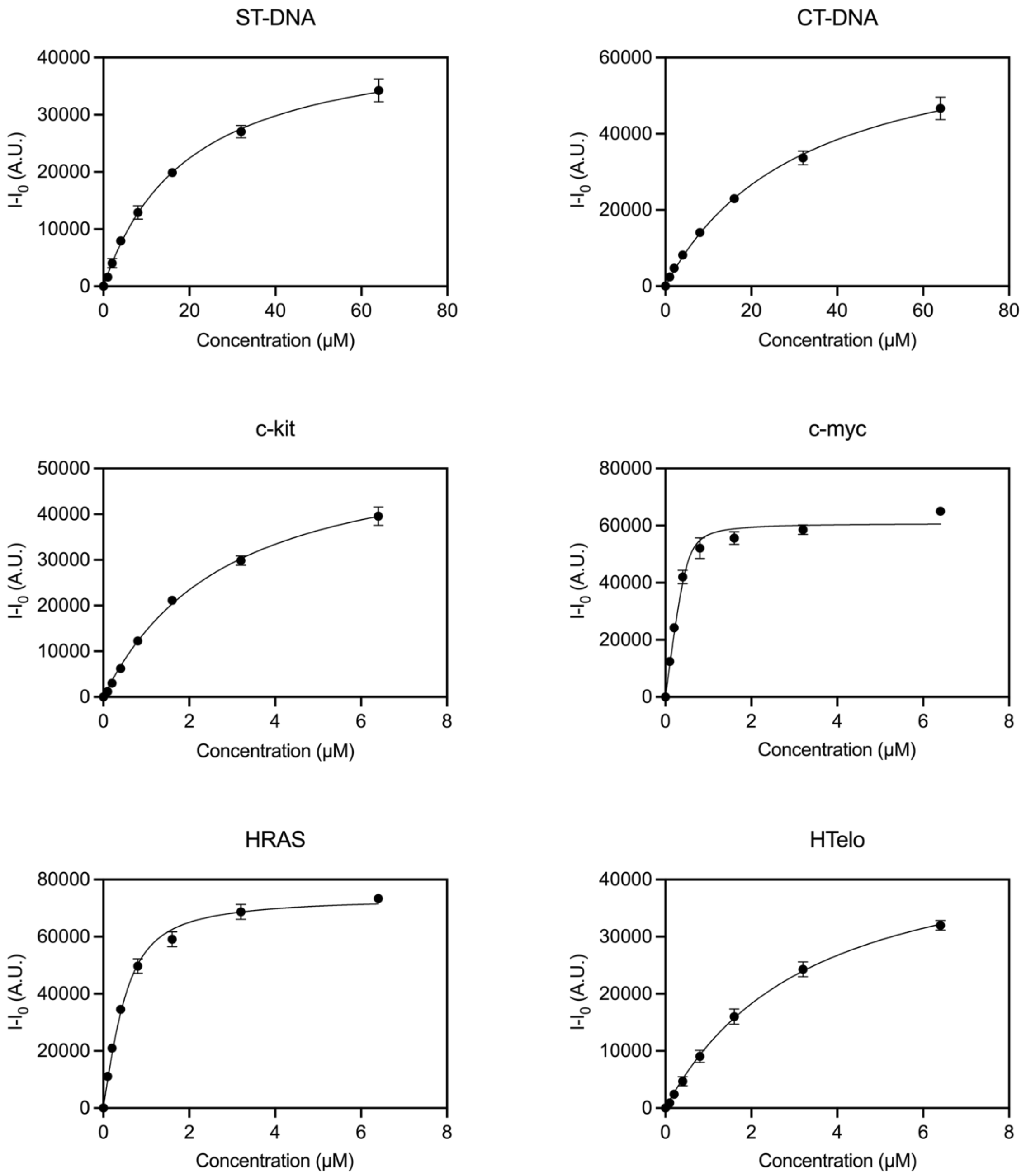

Figure S40. DNA emission titrations with [Pt($\mathbf{L}^2$)] against a range of DNA topologies. Concentration of compound was kept constant at 1 μM and DNA concentrations tested at 0, 0.1, 0.2, 0.4, 0.8, 1.6, 3.2 and 6.4 μM for G4 topologies, and 10x BPE for duplex topologies. Excitation was carried out at 380 nm and emission collected at 520 nm.

Table S5: Calculated binding constants (K_a, μM^{-1}) for Pt(**L**[1]) and Pt(**L**[2])

DNA structure	Pt(**L**[1])	Pt(**L**[2])
c-kit	0.65 ± 0.08	0.20 ± 0.01
c-myc	6.96 ± 2.61	12.73 ± 4.25
HRAS	7.98 ± 4.0	2.28 ± 0.25
HTelo	0.67 ± 0.08	0.17 ± 0.01
ST-DNA	0.64 ± 0.06	0.053 ± 0.003
CT-DNA	0.47 ± 0.05	0.032 ± 0.002

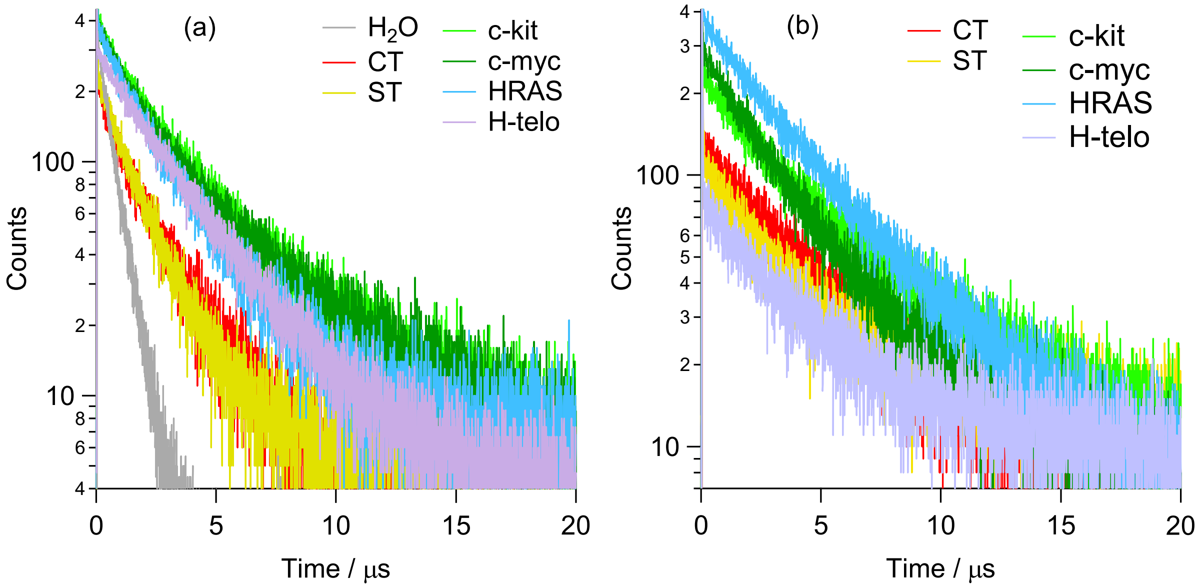

Figure S41. Normalised time-resolved emission decays of (a) [Pt(**L**[1])] and (b) [Pt(**L**[1])] in the absence and presence of double-stranded DNAs (CT, ST) and G4 forming DNAs(c-*Myc*, c-Kit 2, HRAS, H-telo).

Table S6. Intensity-weighted average lifetime (τ_w) in μs corresponding to the data in Figure 2e (*in vitro*). Lifetimes for the probes with DNA, were recorded at 2 μM concentration of probe plus 25 equivalents of G4 and 100 equivalents of duplex DNA, respectively (in buffer pH 7.4)

[Pt(**L**^{1})]						
DNAs	α_1 / %	τ_1 / μs	α_2 / %	τ_2 / μs	τ_w / μs	χ^2
no DNA	100	0.611	-	-	0.611	1.094
CT-dna	34.0	0.458	66.0	2.169	2.001	1.105
ST-dna	71.2	0.026	28.8	1.558	1.496	1.358
C-kit	38.7	0.871	61.3	3.619	3.256	1.159
Cmyc	55.5	1.264	44.5	4.421	3.590	1.308
HRAS	100.0	2.563	-	-	2.563	1.300
H-telo	100.0	2.514	-	-	2.514	1.405

[Pt(**L**^{1})] τ_w / μs	Round 1	Round 2	Round 3	Round 4	Round 5	Averaged	Std. deviations
no DNA	-	-	0.639	0.611	0.617	0.622	0.012
CT-dna	1.729	2.001	-	-	-	1.865	0.136
ST-dna		1.496	1.804	1.743	1.710	1.688	0.116
C-kit	2.576	3.256	-	-	-	2.916	0.340
Cmyc	3.813	3.590	3.005	3.592	3.741	3.548	0.285
HRAS	-	2.563	-	-	-	2.563	-
H-telo	2.697	2.514	2.674	-	-	2.628	0.081

[Pt(**L**^{2})]						
DNAs	α_1 / %	τ_1 / μs	α_2 / %	τ_2 / μs	τ_w / μs	χ^2
no DNA	-	-	-	-	-	-
CT-dna	100.0	4.184	-	-	4.184	1.286
ST-dna	100.0	3.116	-	-	3.116	1.375
C-kit	32.2	1.369	67.8	5.582	5.141	1.102
Cmyc	52.4	2.092	47.6	4.155	3.420	1.273
HRAS	100.0	3.886	-	-	3.886	1.176
H-telo	100.0	3.640	-	-	3.640	1.391

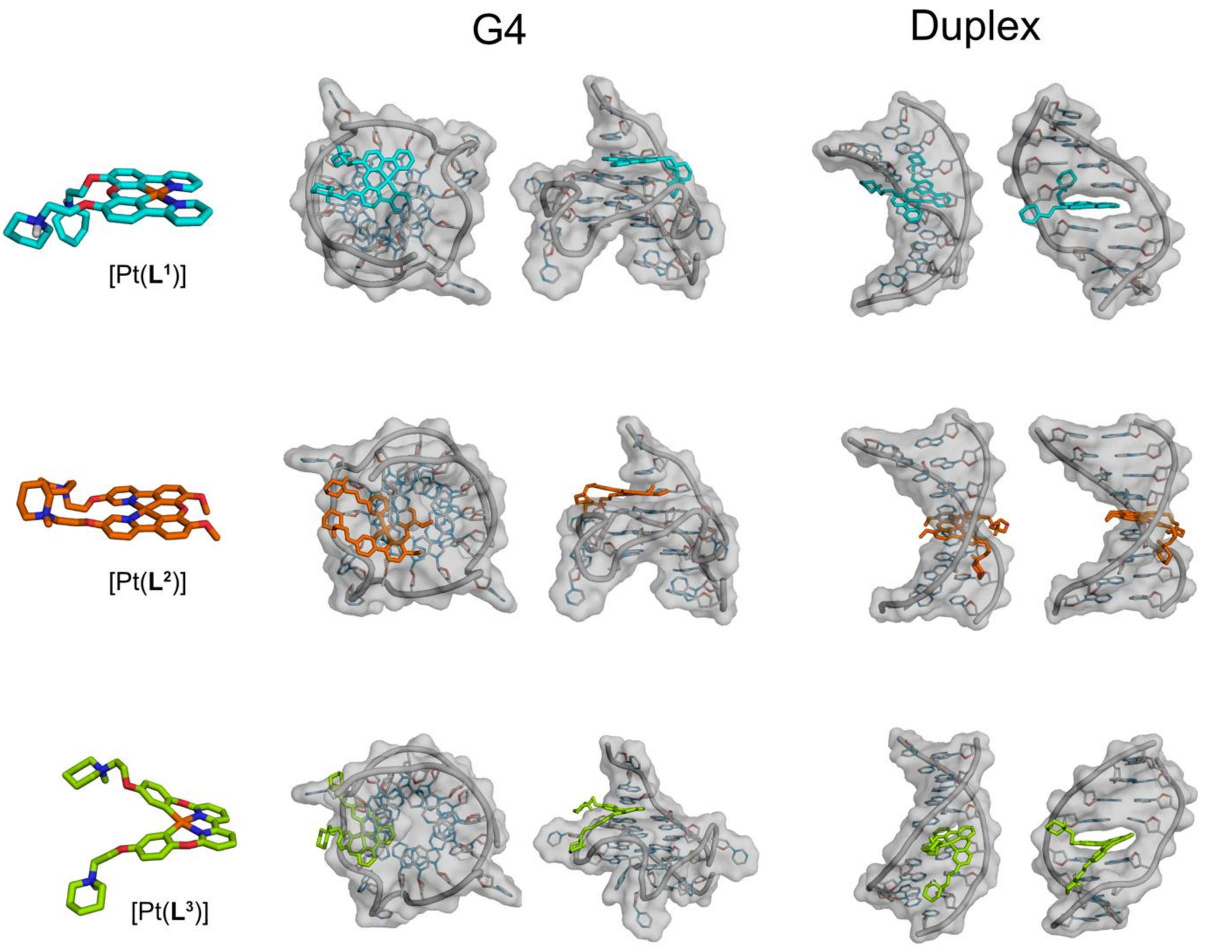

Figure S42. Molecular docking of platinum(II) complexes to G-quadruplex and duplex DNA. Left: DFT-optimized structures of [Pt(L^1)] (cyan), [Pt(L^2)] (orange), and [Pt(L^3)] (green). Centre: Lowest energy docked poses with the c-MYC G-quadruplex (PDB: 2L7V), Right: Lowest energy docked poses with B-form duplex DNA (PDB: 1XRW), DNA shown as grey sticks with transparent surface.

Multiphoton Microscopy data

Live and Fixed Cell experiments

U2OS cells were seeded (3×10^4 cells, 250 µL) on 8-well chambered microscope slides (Lab-Tek Nunc II, part number 155411, ThermoFisher Scientific, Germany) and incubated overnight. For live cell experiments, the growing media (DMEM + 10% Fetal Bovine Serum supplement) was replaced by Phenol Red-free Fluorobrite DMEM media (A1896701, ThermoFisher, Germany) after PBS wash (x2). For fixed cell experiments, cells were washed (x2) in PBS before and after incubation with ice-cold methanol for 8 min. Live and fixed cells were further treated with the compounds under study (5 µM, 100 µL) and visualised under the microscope within 0-6 h at room temperature. For live cells, 5% CO_2 atmosphere was provided by a microscope stage.

Two-photon Confocal imaging

Emission images were collected using an inverted confocal laser-scanning microscope, Leica TCS SP5-II (Leica Microsystems Ltd, Germany) at room temperature. [Pt($\mathbf{L^1}$)] and [Pt($\mathbf{L^2}$)] emission in two emission bands (480 - 580 nm and 610 - 690 nm) was collected using two detectors following two-photon 780 nm excitation from a Chameleon Vision-II Ti:Sapphire laser (Coherent Inc., Germany). A 100x (HCX PL APO CS, N. A. = 1.4, oil immersion, part number 11506210, Leica) objective was used to collect images at 1024 x 1024 pixel resolution. All images were processed in the Leica Application Suite X (LAS X, v.3.7.1) software package.

Phosphorescence Lifetime Imaging Microscopy (PLIM)

Two-photon PLIM imaging was performed using the same microscope system described in the previous section and the same excitation and detection wavelengths. The PLIM data were acquired with an SPC-830 photon counting card and Spcm64 software (both from Becker & Hickl). The resolution of PLIM images was 256×256 pixels with 256 time channels. The IRF was obtained by measuring the second harmonic generation (SHG) signal from crystals of urea. The PLIM data were analysed using the SPCImage software (Becker & Hickl) using 3 x 3 square binning (bin 1) and fitting with a monoexponential function. Nuclei and nucleoli were selected by masks (SPCImage) with further statistical analysis using OriginPro 2024 (OriginLab, USA).

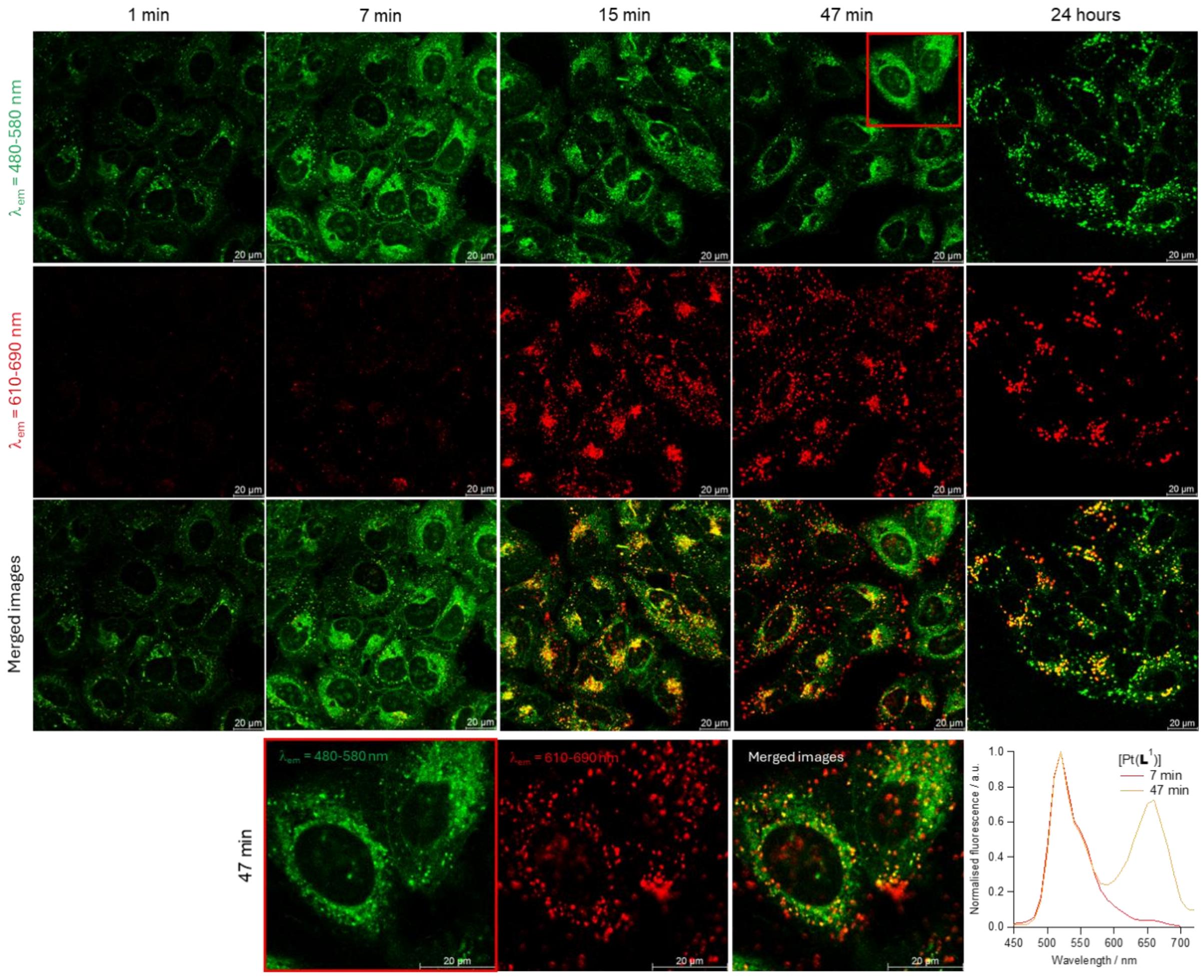

Figure S43. Confocal images recorded for live U2OS cells stained with 5 mM of [Pt(**L**^{1})] at different time points after the dye addition to the media. Images recorded following 780 nm multiphoton excitation and detection in two emission bands: 480-580 nm (monomer) and 610-690 nm (aggregate) regions. Scale bars are 20 μm. The bottom row: Confocal images recorded with higher magnification, 47 min following the incubation with the dye, to illustrate different localisation of signals corresponding to monomeric form (480-580 nm) and aggregated form of [Pt(**L**^{1})] (610-690 nm) within live cells. (Bottom right) Normalised emission spectra recorded at 7 and 47 min following [Pt(**L**^{1})] incubation with cells.

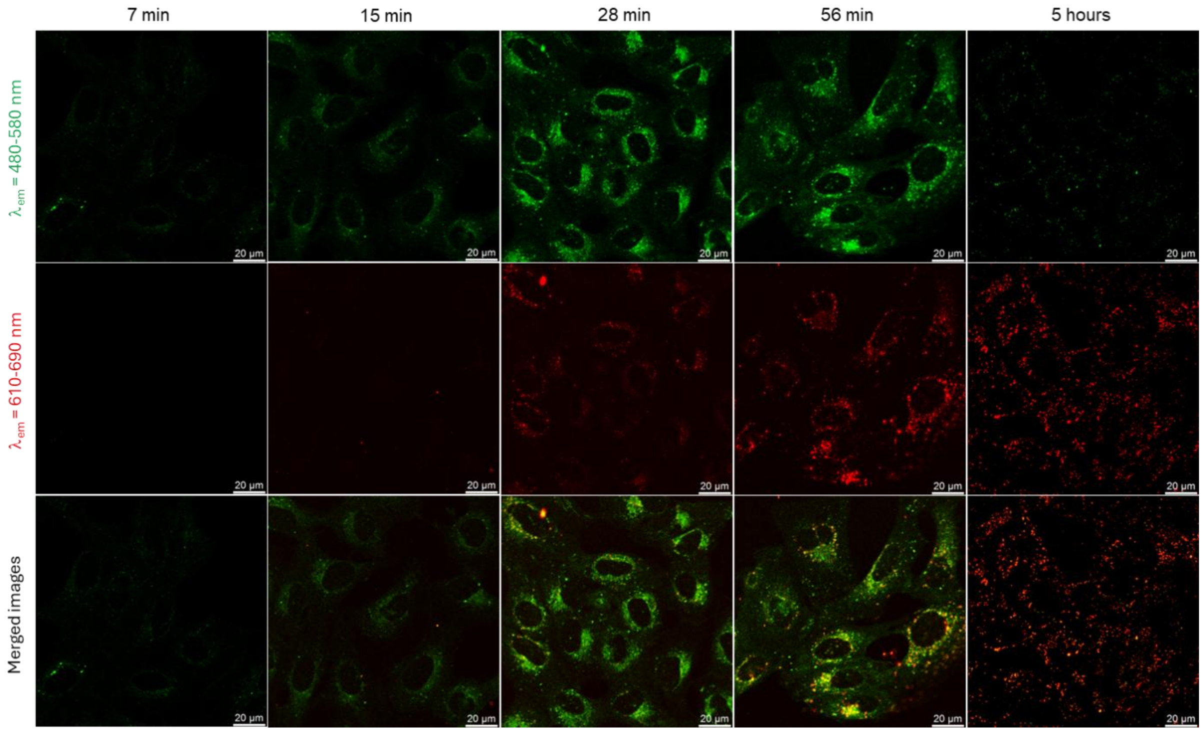

Figure S44. Confocal images recorded for live U2OS cells stained with 5 µM of [Pt(**L**^{2})] at different time points after the dye addition into the media. Images recorded following 780 nm multiphoton excitation and detection in two emission bands: 480-580 nm (monomer) and 610-690 nm (aggregate) regions. Scale bars are 20 µm.

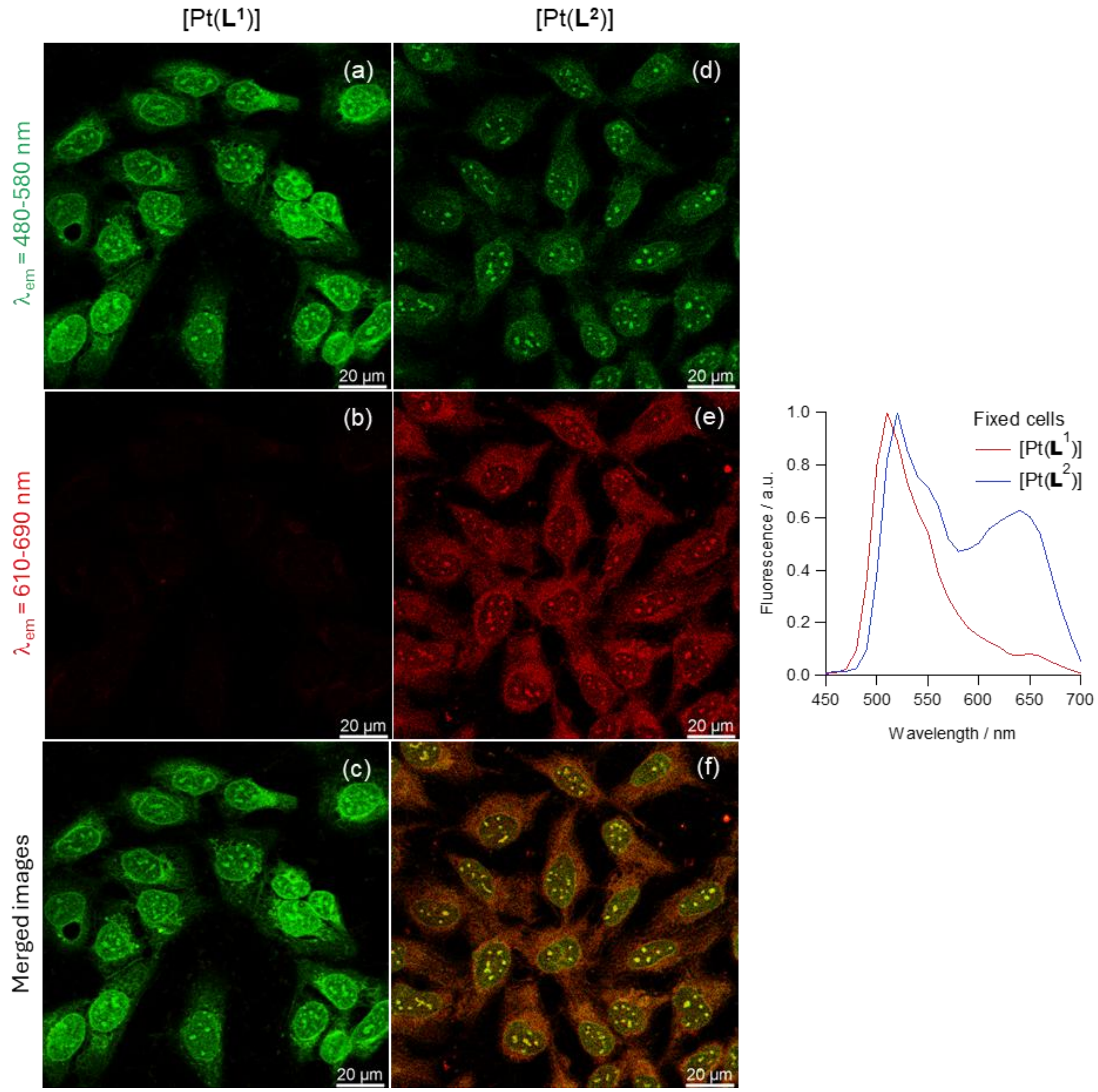

Figure S45. Confocal images recorded for fixed U2OS cells stained with 5 µM of (a-c) [Pt(**L**[1])] and (d-f) [Pt(**L**[2])] following 780 nm multiphoton excitation and detection in two emission bands: 480-580 nm (monomer) and 610-690 nm (aggregate) regions. Scale bars are 20 mm. (g) Emission spectra recorded with cells in (a-c) and (d-f) for [Pt(**L**[1])] and [Pt(**L**[2])], respectively. This spectral data is also presented in Figure 3f of the main text and is reproduced here for convenience.

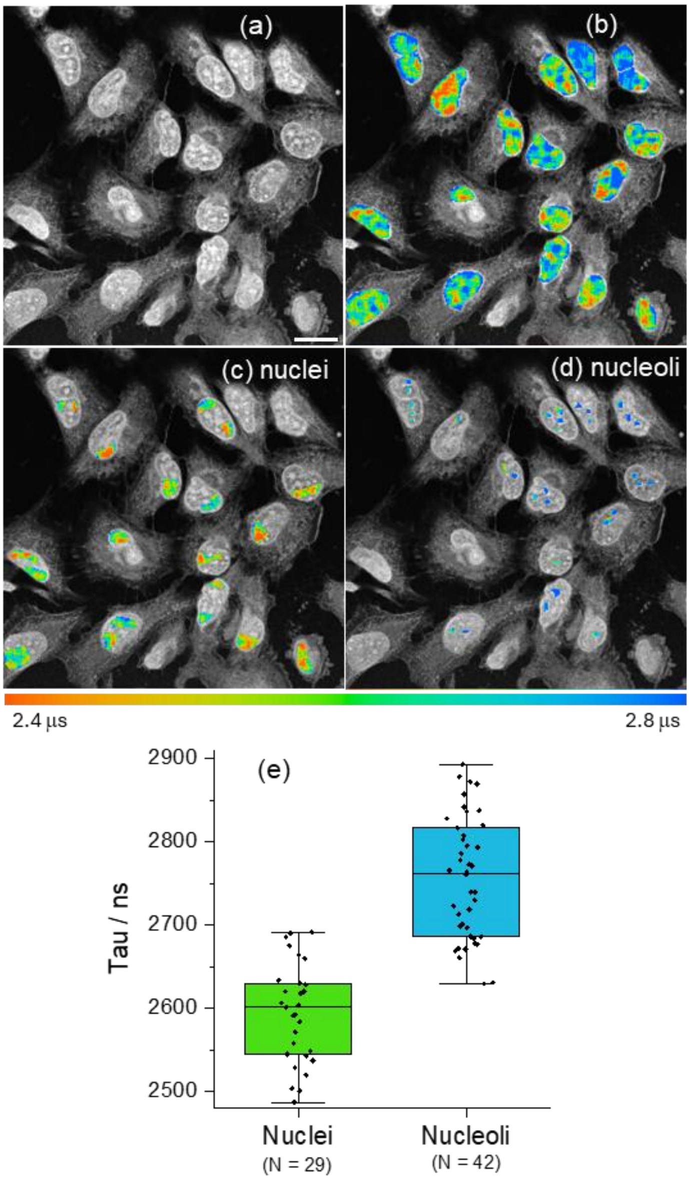

Figure S46. (a) Phosphorescence Intensity and (b-d) lifetime (PLIM) images recorded with fixed U2OS cells stained with 5 µM of [Pt(**L**[1])] following 780 nm multiphoton excitation and detection in the 480-580 nm region. Scale bar is 20 µm. (c,d) Segmented PLIM images with segmentation of (c) part of nuclei free of nucleoli and (d) nucleoli; (e) statistical analysis of phosphorescence lifetimes in those regions (t), $p<0.05$ for all condition pairs.

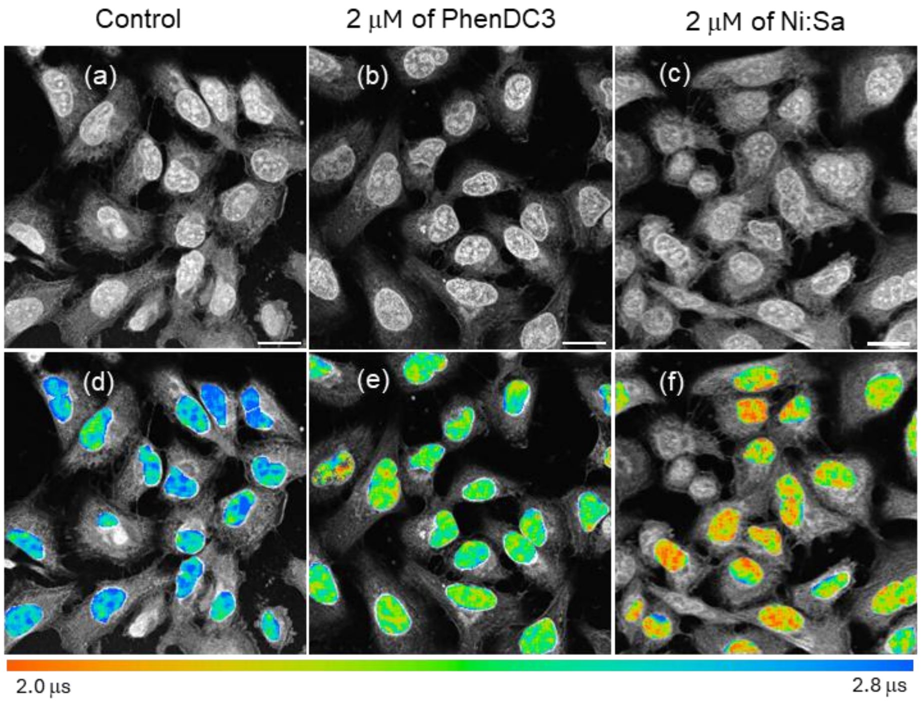

Figure S47. (a-c) Phosphorescence Intensity and (d-f) lifetime (PLIM) images recorded with fixed U2OS cells stained with 5 μM of [Pt(**L**[1])] following 780 nm multiphoton excitation and detection in the 480-580 nm region. Scale bars are 20 mm. Images for the sample treated with (b,e) PhenDC3 (2 μM) and (c,f) Ni-Salphen (2 μM).

References

1. M. A. Soto, V. Carta, R. J. Andrews, M. T. Chaudhry, M. J.MacLachlan, *Angew. Chem. Int. Ed.* **2020**, *59*, 10348–10352.
